# Assessment of trachoma in suspected endemic areas within 16 provinces in mainland China

**DOI:** 10.1371/journal.pntd.0007130

**Published:** 2019-01-28

**Authors:** Jialiang Zhao, Silvio Paolo Mariotti, Serge Resnikoff, Yuqin Wang, Shicheng Yu, Mingguang He, Yingchuan Fan, Haidong Zou, Wenfang Zhang, Yading Jia, Lihua Wang, Huaijin Guan, Xiao Xu, Leilei Zhan, Lei An, Quanfu Ye, Ningli Wang

**Affiliations:** 1 Department of Ophthalmology, Peking Union Medical College Hospital, Chinese Academy of Medical Sciences, Beijing, China; 2 Non Communicable Diseases and Mental Health, World Health Organization, Geneva, Switzerland; 3 Brien Holden Vision Institute and SOVS, University of New South Wales, Sydney, Australia; 4 Pharmacy Department, Xuanwu Hospital of Capital Medical University, Beijing, China; 5 Health Statistics Department, Chinese Center for Disease Control and Prevention, Beijing, China; 6 Centre for Eye Research Australia, University of Melbourne, Melbourne, Australia; 7 Department of Ophthalmology, Sichuan Provincial People’s Hospital, Chengdu, China; 8 Department of Ophthalmology, Shanghai First People's Hospital, Shanghai Jiaotong University, Shanghai, China; 9 Department of Ophthalmology, The Second Hospital of Lanzhou University, Lanzhou, China; 10 Department of Ophthalmology, Shanxi Eye Hospital, Taiyuan, China; 11 Department of Ophthalmology, Shandong Provincial Hospital affiliated to Shandong University, Jinan, China; 12 Eye Institute, Affiliated Hospital of Nantong University, Nantong, China; 13 Rehabilitation Administration Department, National Institute of Hospital Administration, National Health and Family Planning Commission, Beijing, China; 14 Beijing Institute of Ophthalmology, Beijing Tongren Hospital, Beijing, China; RTI International, UNITED REPUBLIC OF TANZANIA

## Abstract

**Background:**

China used to be among the countries with a high prevalence of trachoma. At the launch of The Global Elimination of Trachoma (GET) 2020 campaign by the World Health Organization (WHO) in 1996, China was placed on the list of countries endemic for trachoma based on historical data. However, empirical observation and routinely collected eye care data were suggesting that trachoma was no longer a public health problem. To determine whether the GET 2020 goals had been met in P. R. China, we conducted a targeted assessment with national scope.

**Methodology/principal finding:**

Province assessment teams, trained in WHO Trachoma Rapid Assessment (TRA) methodology and in WHO simplified trachoma grading system, carried out assessments in 16 provinces (among them, 2 provinces conducted pilot assessment). Based on the published literature, including national and international reports, suspected trachoma-endemic areas within each province were identified. Within these areas, trachomatous inflammation- follicular (TF) assessments were carried out in at least 50 grade-one children in primary schools serving villages with the lowest socio-economic development. Trachomatous trichiasis (TT) and corneal opacity (CO) assessments were conducted among persons aged 15 and over in villages within the catchment area of the selected schools. Of 8,259 children examined in 128 primary schools in 97 suspected trachoma endemic areas, only 16 cases of conjunctivitis were graded as TF. 38 cases with TT were found among the 339,013 examined residents in villages surrounding the schools. Among these 97 suspected trachoma endemic areas in only three was the prevalence of TT more than 0.2%.

**Conclusions/significance:**

This large study suggested that trachoma was not a public health problem in 16 provinces that had been previously suspected to be endemic. These findings will facilitate planning for elimination of trachoma from PR China.

## Author summary

China used to a country with a high burden of trachoma, but recent empirical observation and routinely collected eye care data were suggesting that elimination of trachoma had been achieved in China. In order to verify that and reach the target set in the WHA 51.11 Resolution, we have assessed current situation using the World Health Organization’s Simplified Trachoma Grading System and TRA. As China is a country with a big population and huge regional disparity, this national condition is also considered in methodology design and implement. Among 8,259 children examined in 128 primary schools in 97 suspected trachoma endemic areas, only 16 cases of conjunctivitis were graded as TF. 38 cases with TT were found among the 339,013 examined residents in villages surrounding the schools, among these 97 suspected trachoma endemic areas only in 3 areas, the prevalence of TT was more than 0.2%. Trachoma is no longer a public health issue in China, however, we still need to pay attention to TT in the remote and poor areas. Therapeutic interventions for few residual patients have been provided for free, and health education and publicity on trachoma prevention have been continued in the whole society.

## Introduction

China used to be among the countries with a high prevalence of trachoma. Since the new China was founded in 1949, the government has actively supported trachoma control activities. In 1960s, the Chinese government adopted a policy to strengthen healthcare provision in rural areas, which in turn provided an opportunity to increase trachoma control efforts in rural areas. Several epidemiological surveys in the 1990’s showed that the prevalence of trachoma had been drastically decreased from earlier years and its severity reduced in terms of trachomatous trichiasis cases reported in medical records. According to those surveys, trachoma prevalence among primary and secondary school students was 16% and 18%, respectively, in 1992; dropping to 11% and 14% in 1995, and to 8% and 8% in 2000 [1].

In 1999 at the invitation of the Chinese Ministry of Health, the World Health Organization (WHO) organized a national workshop on trachoma control to review the status of the disease and accelerate the elimination of blindness from trachoma in China [2]. Since then, prevention of blindness activities in China was implemented by the Chinese government and international partners (e.g. The Lions Clubs International). Surgical service for cataracts and elimination of trachoma were among the priorities. The number of trachoma cases identified in public health activities, in surveys on causes of visual impairment and in clinical care services were steadily decreasing–consistent with a rapid socio-economic development, significant improvement of personal hygiene (particularly in schools), as well as increased access to eye care service across China. The China Nine-Province Survey in 2006 [3,4], the Second National Survey on Disabled Persons in 2006 and other data provided evidence that trachoma was no longer a common cause of vision loss [5,6], despite some articles reporting the finding of active trachoma cases in schools [7,8].

At the launch of the Global Elimination of Trachoma (GET) 2020 campaign by the WHO in 1997, China was included on the list of the countries to be verified for possible presence of trachoma based on historical epidemiological data [9]. Although data from blindness surveys and routinely collected clinical data were showing that blindness from trachoma was no longer a public health issue, it was decided to assess the current situation using internationally adopted epidemiological tools, definitions and standards, including the WHO Simplified Trachoma Grading System [10]. The thresholds for elimination of trachoma as a public health problem include: i.e. 1) TF prevalence of <5% in children 1–9 years; 2) prevalence of TT in people aged 15 years or more of <0.2%; and 3) evidence that the health system is able to identify and manage incident TT cases [11].

Despite current recommendations on population-based prevalence surveys, TRA was used in this survey in view of the enormity of such a task in the most populous country China. In 2012, the former Chinese National Health and Family Planning Commission, with WHO technical support and funding from Lions Clubs International, undertook the assessment of the elimination of trachoma in P.R. China. By the end of the year 2015, the assessment was completed and this article reports main results.

## Methods

The assessment of the status of elimination of trachoma in China followed the recommendation set forth in World Health Assembly resolution 51.11, i.e. the use of the TRA [14]. In summary, for each selected province, TRA was used to determine if trachoma was present, and if trachoma was above the threshold of 4 children with TF per school, then a population based prevalence survey would be carried out. The period for recruitment was nearly 3 months, duration for assessment was about 18 months, and data collection and cleansing was finished for about 3 months.

### Selection of provinces

TRA was conducted in all provinces historically known, or suspected of having trachoma-endemic areas. Identification of the provinces to be surveyed was based on two approaches: a) Identification based on published historical data and reports on trachoma. Our study fully considered those existing studies and papers, but the publication dates of those were different, and survey strategy and definition standards used were substantially different (e.g. the Chinese trachoma grading system vs. the WHO grading system). In our study, with the support of WHO experts, we adopted for the first time the WHO international trachoma grading standard. Regarding our selection of survey location, we started from the Report of the first meeting of the WHO alliance for the global elimination of trachoma published in 1997. In this workshop, representatives from all provinces in China reported the historic and current information about epidemic condition of trachoma. Based on that report [2], there were 12 provinces with suspected trachoma endemic areas–Hebei, Inner Mongolia, Liaoning, Anhui, Henan, Hainan, Guizhou, Yunnan, Shaanxi, Gansu, Qinghai and Ningxia. b) Identification based solely on low socio-economic status and access to water and sanitation. This led to the addition of 2 autonomous regions (organizationally equivalent to provinces)—Guangxi and Tibet—based on ranking among the 5 provinces with the lowest gross domestic product (GDP) and per capita income in 2013.

Additionally, 2 provinces—Shandong and Sichuan—were included as pilot assessment provinces to examine our method of study. After the pilot test, our methodology was improved and perfected. TRA was also conducted in these pilot assessment provinces, however the results should be presented separately.

### Selection of suspected trachoma endemic areas

Each province was divided into several geographic areas with a population of approximately 100,000 to 150,000, using county and township boundaries and census data. Suspected trachoma endemic areas within these geographic population clusters were determined in the following ways: a) Evidence regarding the presence of trachoma based on the published literature; b) Existing data on trachoma among primary-school students over the past five years: c) Existing routinely-collected data on trachoma found in patients presenting to eye care services.

TRA was carried out in selected primary schools in the identified suspected endemic areas within each of the selected provinces and included: a) An assessment for TF in at least 50 grade-one school children within the surveyed areas. B) An assessment of TT and CO in persons aged 15 years or over in villages within the catchment area of the surveyed schools.

### Trachoma diagnosis and grading

The WHO simplified Trachoma Grading System [10] was used to provide consistency with internationally agreed approaches for trachoma assessment and with criteria set by the WHO Alliance for the Global Elimination of trachoma by 2020 [12]. Case finding focused on three grades of trachoma: TF, five or more follicles more than or equal to 0·5mm on the upper tarsal conjunctiva; TT, at least one ingrown eyelash touching the globe due to trachoma, or evidence of epilation; CO, corneal opacity blurring part of the pupil margin due to trachoma [13].

### Training provincial survey teams

In each selected province, 2 or 3 provincial survey teams were assembled, equipped and trained. Each provincial team included 3 certified ophthalmologists, who were ultimately responsible for trachoma grading in the assessment. Each ophthalmologist was equipped with a head-mounted magnifier (×2·5), torch, and alcohol-based hand gel for sanitizing hands.

Ophthalmologists of each survey team attended a national two-day structured training course in Beijing first, then a two-day field training in a county in Inner Mongolia autonomous region, where more TF cases may existed according to local ophthalmologist’s opinion. The national-level trainers included a WHO officer and international trachoma experts. Training included the WHO SAFE strategy, the WHO TRA methodology, the WHO Simplified Trachoma Grading System, laboratory confirmation of Chlamydia trachomatis as well as procedures for identification of population to be surveyed.

This training included tests-of-agreement for trachoma identification and grading between each trainee and the WHO trachoma experts. All trainees entered into 2 classrooms and examined 80 schoolchildren, who were healthy, had conjunctivitis or were real trachoma cases (only 2 positive TF cases existed in each classroom, since we hardly to find TF cases. In fact, we collected TF cases in several primary schools in Inner Mongolia together for this agreement-test). Each team member was observed by a WHO expert grader who assessed agreement. All 54 trainees passed the agreement testing as they were measured totally equivalent to the WHO expert grader.

After academic and field training at the national level, two-day provincial-level training was organized in each province to educate local ophthalmologists and supporting staff regarding TRA process [13]. Ophthalmologists who attend the national training course were the trainers. However, only the ophthalmologists who attend the national training course are responsible to perform the examination, trachoma diagnosis and grading in the TRA assessment. The purpose of provincial training was to equip local ophthalmologists and supporting staff professional and standardized method in the TRA assessment.

The national and provincial training course included the detection of TT and CO. The diagnostic criteria of TT and CO are relatively easy for ophthalmologists. However, trichiasis may be caused by other situations, such as senile or spasmodic trichiasis, CO may be caused by viral or bacterial infection. Thus, we emphasize to do differential diagnosis in training and in the assessment. If the person is absence of TS, it may indicate that the trichiasis or CO was not trachomatous in origin. Since in the training practice in Inner Mongolia, there are too few cases of TT and CO, thus, we didn’t perform TT and CO agreement test.

### Hospital records review

The trained provincial survey teams began field work by visiting ophthalmologists in county or local leading hospitals within the suspected endemic areas to discuss their trachoma knowledge and enquire about TF diagnoses or TT surgeries performed in recent years. Hospital’s records were also checked regarding diagnosis of corneal blindness due to trachoma.

The TRA was carried out in the identified suspected endemic areas within each of the selected provinces and included: a) An assessment for TF in at least 50 grade-one school children within the surveyed areas. B) An assessment of TT and CO in persons aged 15 years or over in villages within the catchment area of the surveyed schools.

### Assessment of TF in grade-one school children in TRA

Even in the complete absence of evidence or local awareness regarding trachoma cases, one or more primary schools with the worst socio-economic conditions, worse water supply and poorest schooling conditions were selected for TRA in each of the selected areas. Priority was given to boarding schools with a high enrollment of children from rural families as trachoma is easily transmitted in centralized environment. There was no gender bias as quantities of male students and female students in each school were very close. At each selected school, survey team was mandated to examine at least 50 grade-one students (usually seven-year-old). School teachers were fully mobilized to encourage students and their family members to participate to address potential sources of bias.

**Fig 1:**
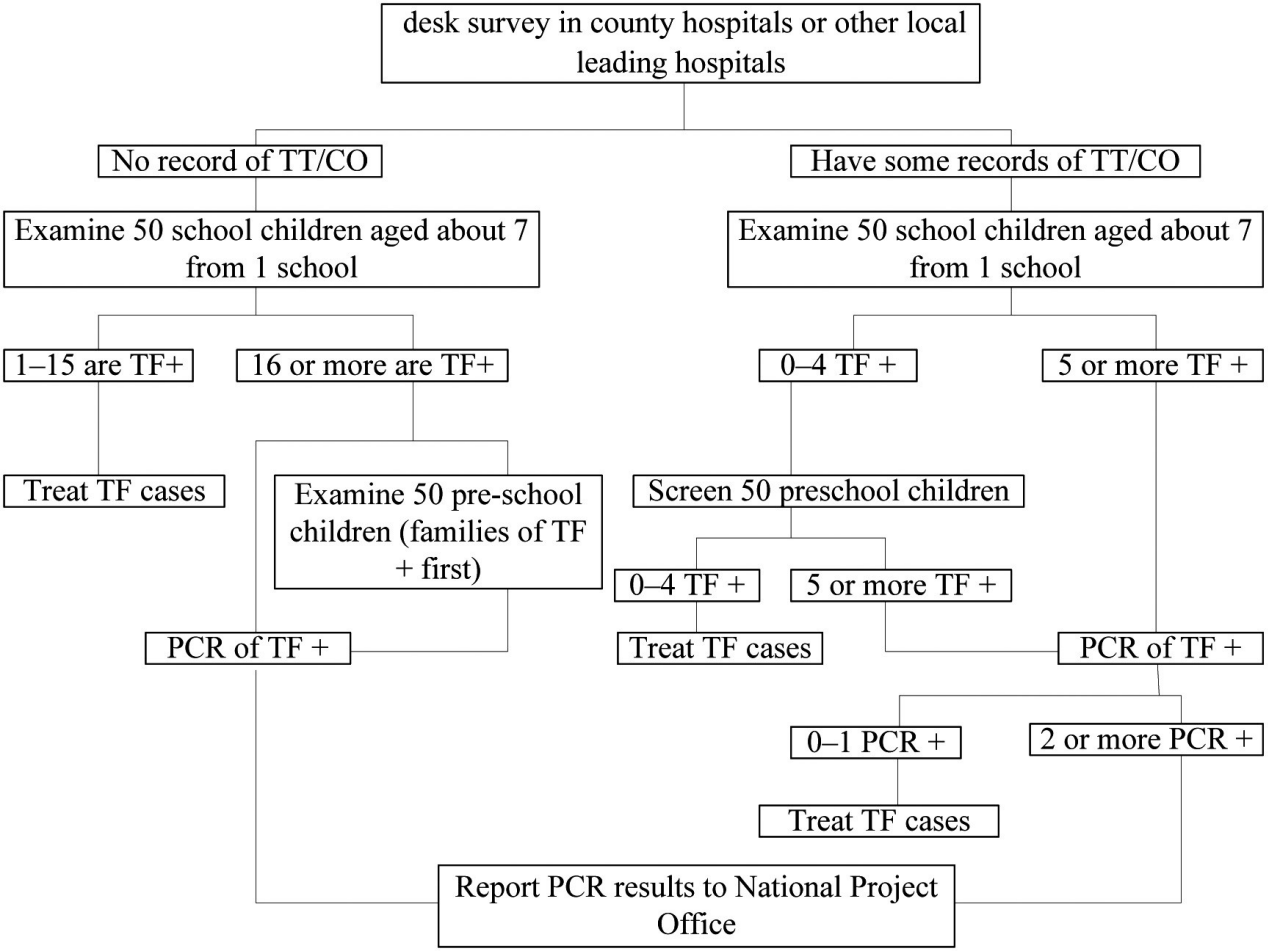
Flow path for TRA.

In order to reduce the risk of missing possible endemic areas, two alternative strategies shown in Fig 1 were used in assessing the presence and detailed number of TF in the selected schools, depending on whether TT or CO were found in the desk review of hospital records within the suspected endemic area. The TRA approach was used when no records of TT or CO were found in any of the hospital reviews. The second strategy was used in schools when one or more records of TT or CO were found in any of the surveyed hospitals.

### Assessment of TT and CO in village residents in TRA

TRA for TT and CO was conducted in two ways: a) If recorded cases of TT or CO were found during the review of patient records in any of county/lead hospitals within suspected endemic areas, these individuals were contacted for reexamination/confirmation and the affected village(s) was screened for additional cases of TT or CO among residents (defined as people living in the village for the last six months) aged 15 years or older; b) If TT or CO cases were not found during hospital records reviews, survey team visited at least one village surrounding the school assessed for TF to look for TT or CO among residents aged 15 years or older. In both assessment scenarios, known or suspected TT or CO cases were identified by consulting village leaders and doctors, followed by a visit to the home of the TT or CO suspect to confirm the diagnosis. Village leaders and doctors after standardized training were fully mobilized to participate as assistants for the assessment work in the village and inspection rate was guaranteed to address potential selection bias. The village leaders and doctors were familiar with every family and residents in the village, since the population of villages is usually less than 1,000.

After the assessment for TT and CO in suspected endemic areas, an approximate prevalence rate for TT and CO in the area was calculated as the number of confirmed cases divided by the estimated number of persons aged 15 or above in the village.

### Population-based assessment of TF, TT and CO

As data from each suspected areas became available, the national project office analyzed the findings to decide whether a population-based survey was needed. According to the project plan, a population-based survey was considered warranted if more than 4 TF cases were identified among the 50 children examined at any one school. Because there was no school with more than 2 TF cases, a population-based survey for TF was not undertaken in any of the suspect endemic areas.

If an area was found to have more TT or CO cases, we should expand survey area. If the prevalence (unadjusted by sex and age) of TT or CO is higher than the threshold 0.2%, the assessment was also conducted in nearby areas. If the prevalence of TT or CO was confirmed more than the threshold 0.2%, then this area needs to conduct the population-based survey for TT or CO.

After TRA, we just found a few TT cases. For further confirming the situation of TT and getting more information of TT, we mobilized local ophthalmologists who attended provincial training to conduct a population-based survey for evaluating the situation of TT more broadly.

### Intervention for confirmed trachoma cases

For TF-positive children a provision of drugs for treatment was secured through county resources. The treatment regimen was the one recommended by WHO GET2020 campaign–one-gram of oral azithromycin, either tablet or suspension, for adults and 20 mg per kg body weight for children. Family members of TF patient were also to be treated.

For TT cases, a bilamellar tarsal rotation procedure or other similar oculoplastic method was provided free of charge to the patient. Electrolysis was used for the patients with one or two inverted eyelashes touching the globe (not the cornea) in the absence of entropion. County hospitals were selected to provide the required surgery.

### Quality control

Strict quality control measures were undertaken: a) The project design was based on WHO standards for trachoma control (TRA and Simplified Grading System). b) A pilot study was undertaken to verify the feasibility of the planned assessment methodology. c) National and provincial training sessions were held for survey personnel with formal inter-observer agreement testing of grading consistency. d) Supervisory activities were implemented throughout the field assessment process with monitoring and participatory supervision by national and international experts.

### Data management

Each provincial survey team had a dedicated analyst responsible for data recording, computerized data entry, and data review for completeness and accuracy. EpiData 3.0 was used for data entry and analysis.

### Ethical review

This study basically adopted routine public health measures. All involved in the intervention were provided with written informed consent which asked participants themselves or juveniles’ parents to sign on it. IRB of Peking Union Medical College Hospital approved the ethical review of elimination of blinding trachoma by 2016 in China on Feb. 6, 2013. The statement shown in the certificate is “this project is designed scientifically, risks and benefits of human subjects are rational, and letter for informed consent meets the ethical requirement”. All data analyzed were anonymized.

**http://dx.doi.org/10.17504/protocols.io.qu9dwz6**

## Results

### The Information from the pilot study

From June to September 2013, Shandong and Sichuan provinces were selected to conduct pilot assessment. Table 1 showed the information from the pilot assessment.

**Table 1:** The information of TF and TT from the pilot assessment.

Province (No. of Suspected Areas)	Location of Suspected Area	TF		TT			
		No. of Schoolchildren Examined	No. of TF cases	No. of villages assessed	No. of villagers Examined	No. of Villagers with TT	Prevalence of TT
Shandong (7)	Tongjing Town of Yinnan County, Linyi City	176	0	1	1,345	0	0·000%
	Mazhuang Town of Feixian county, Linyi city	100	0	2	1,500	0	0·000%
	Qingshui Town of Guanxian County Dezhou City	105	0	5	475	0	0·000%
	Suliuzhuang Town of Xiajin County, Dezhou City	106	0	5	4,725	1	0·001%
	Deping Town of Linyi County, Liaocheng City	100	0	5	2,275	0	0·000%
	Wangfeng Town of Laixian County, Liaocheng City	100	0	5	5,975	0	0·000%
	Taishan District of Tai'an City	200	0	2	1,885	0	0·000%
Sichuan (7)	Danshan Town of Ziyang City	50	0	2	1005	14	1.393%
	Cotton Slope Town of Naxi District,Luzhou City	50	0	1	102	0	0·000%
	Jiangyang District of Luzhou City	50	0	1	111	0	0·000%
	Sijia Village of Zhaojue County,Liangshan Yi Autonomous Region	50	0	1	103	0	0·000%
	Ningnan County of Liangshan Yi Autonomous Region	50	0	2	116	2	1.724%
	Wuyi Town of Xiangtang County,Aba Tibetan Autonomous Region	50	0	3	600	0	0·000%
	Xinminchang Town of Pixian County	50	0	2	1300	0	0·000%
TOTAL (14)		1237	0	37	21517	17	0.079%

7 suspected areas were identified in Shandong provinces, and 887 students aged about 7 from 14 primary schools were examined for TF. No TF cases were found. 18,180 villagers aged 15 or above from 25 villages nearby the selected primary school were examined for TT or CO. Only 1 TT case and no CO case were found.

7 suspected areas were identified in Sichuan province, and 350 students aged about 7 from 7 primary schools were examined for TF. No TF cases were found. 3,337 villagers aged 15 or above from 12 villages nears the selected primary school were examined for TT or CO. 1 CO case was found, however, 14 TT cases among 1005 persons in one area and 2 TT cases among 116 persons in one area were found, the prevalence was 1.393% and 1.724% respectively. The prevalence was unadjusted for age and gender.

### TF in grade-one school children in TRA

A total of 83 suspected trachoma-endemic areas were identified in those 14 provinces in mainland China. Using TRA methodology at least 50 grade-one children (generally seven years of age) in each of 107 schools were assessed for TF. 7,022 children received the assessment, only 16 cases of TF were found in 14 schools, in which TF cases were found in only 2 schools were 2 cases found; in all of the other schools had only 1 case. (Table 2) No TF cases were found in 93 schools among 107 schools,. The threshold of four TF-positive children for further epidemiological investigation and PCR testing for Chlamydia trachomatis was found nowhere.

**Table 2:** TRA result for TF and TT in suspected trachoma endemic areas in 14 provinces.

Province (No. of Suspected Areas)	Location of Suspected Area	TF			TT			
		No. of School examined	No. of Schoolchildren Examined	No. of Children with TF	No. of villages assessed	No. of villagers Examined	No. of Villagers with TT	Prevalence of TT
Hebei (3)	Laowu Village of Pingxiang County, Xingtai City	1	50	0	1	1100	0	0·000%
	Zhouwo Village of Wuqiang County, Hengshui City	1	50	0	1	856	0	0·000%
	Gekan Village of Luanxian County, Tangshan City	1	50	0	1	2180	0	0·000%
Inner Mongolia (9)	Alihe Town of Elunchun Autonomous County, Hulun Buir City	1	127	1	2	1580	0	0·000%
	Sandaowa Village of Liujiazi County, Kulun County Tongliao city	1	132	0	2	253	0	0·000%
	Bayanchagan Town of Keshiketeng County, Chifeng City	1	63	1	2	285	0	0·000%
	Balagergaole Town of Xiwuzhumuqin County, Xilin Gol League	2	439	0	2	560	1	0·179%
	Baochang Town of Taipusi County, Xilin Gol League	1	99	0	2	653	2	0·306%
	Tieshagai Town of Chahar County, Wulanchabu City	1	62	0	2	68	0	0·000%
	Kekeligeng Town of Wuchuan County, Hohhot City City	1	68	0	2	220	0	0·000%
	Shibao Town of Darhan Muminggan Joint Counties, Baotou City	1	76	0	2	456	0	0·000%
	Delingshan Town of Urad County, Bayan Nur City	1	87	0	2	348	0	0·000%
Liaoning (5)	Xifeng County of Tieling City	1	50	0	2	200	0	0·000%
	Benxi Manchu Autonomous County, Benxi City	1	160	0	2	400	0	0·000%
	Jianping county of Chaoyang City	2	100	0	2	800	0	0·000%
	Fushun County of Fushun city	1	50	0	2	200	0	0·000%
	Kuandian Manchu Autonomous County, Dandong City	1	50	0	2	200	0	0·000%
Anhui (7)	Fuxiao Village of Dingyuan County, Chuzhou City	1	50	0	1	4200	0	0·000%
	Longtai Village of Taihe County, Fuyang City	1	50	0	1	4100	1	0·024%
	Daxi Village of Lingquan county, Fuyang City	1	50	0	1	5302	1	0·019%
	Shuangduiji Village of Suixi County, Huaibei City	1	50	0	1	4460	1	0·022%
	Dinggou Village of Sixian County, Suzhou City	1	50	0	1	5400	2	0·037%
	Toutuo Village Yuexi County, Anqing City	1	50	0	1	1895	1	0·053%
	Yaocun Village of Langxi county, Xuancheng City	1	50	0	1	3668	0	0·000%
Henan(4)	Shuikui Village, Guandu Bridge Village and Cangzhai Village of Zhongmou County, Zhengzhou city	1	58	0	3	8975	0	0·000%
	Hongbu Township Subdistrict, Hongbu Village and Yingshui Village of Gushi County, Xinyang City	1	50	0	3	15236	0	0·000%
	Puligudui Village, Licaiyuan Village of Fanxian County, Puyang City	1	53	0	2	5200	0	0·000%
	Qiuzhuang Village and Wuzhuang Village of Xiawa Town, Sheqi County Nanyang City	1	55	0	2	6987	0	0·000%
Hainan (11)	Tianzhong Village and Zhongsha Village of Dongfang City	1	50	0	2	3105	0	0·000%
	Angwai Village and Wanche Village of Ledong County	1	53	0	2	5092	0	0·000%
	Maodao Village and Red Luck Village of Five Fingers Group City	1	50	0	2	2420	0	0·000%
	Damei Village Committee, Tianji Village Committee, Damei Second Village of Liugong Town, Baoting County	1	50	0	3	3745	0	0·000%
	Nanxun Village Committee and Naji Village of Baisha County	1	50	0	2	1980	0	0·000%
	Yalao Fourth-Group Village and Fifth-Group Village of Changjiang County	1	53	0	2	350	0	0·000%
	Nanchao Village and Kunlong Village of Lingao County	1	50	0	2	450	0	0·000%
	Meisuo Village of Lingao County	1	50	0	1	2300	0	0·000%
	Xincunshang Village and Xincunxia Village of Qiongzhong County	1	50	0	2	288	0	0·000%
	Qiaopo Village Committee of Wenchang City	1	50	0	1	1800	0	0·000%
	Shibi Village Committee of Qionghai City	1	50	0	1	4000	0	0·000%
Guizhou (6)	Wanzhai Village and Peace Village of Danzhai County, Dongnan State Guizhou Province	1	52	1	2	2218	2	0·090%
	Yuwa Village and Yanxia Village of Zhenning County, Anshun City	1	50	0	2	3962	1	0·025%
	Stalagmite Village and Star Village of Daozhen County, Zunyi Area	1	57	0	2	6234	3	0·048%
	Huabao Village and Fenghua Village of Songtao County, Tongren Rea	1	72	0	2	4351	1	0·023%
	Gucheng Village and Bangao Village of Sandu County, Qiannan State	1	65	0	2	2776	0	0·000%
	Lixiu Village and Kongming Village of Zhenfeng County, Southwest State Guizhou	1	79	0	2	3195	2	0·063%
Yunnan (8)	Gudi Village of Binzhou County, Dali City	3	169	0	4	7610	0	0·000%
	Big River Town of Fuyuan County, Qujing City	1	112	0	4	7700	0	0·000%
	Ganhai Village of Daguan County, ZhaoTong City	1	70	0	4	9000	0	0·000%
	Unity Village Committee of White River Town, Pingbian County	2	149	2	5	3900	0	0·000%
	Xingjie Village Committee of Xingjie Town, Xishou County	1	183	0	2	5000	0	0·000%
	Naha Town of Mojiang County	2	140	0	3	12948	0	0·000%
	Bangbing Town of Shuangjiang County	2	170	0	4	18100	0	0·000%
	Jiangbian Town of Yuanmou County, Chuxiong State	2	162	0	4	3983	0	0·000%
Shaanxi (4)	Kengzhen Village of Jiaxian County, Yulin City	1	50	0	2	1200	0	0·000%
	Seven-mile Village of Yanchang County, Yan’an City	1	50	0	2	3000	0	0·000%
	Yecun Town of Shangzhou District, Shangluo City	1	50	0	2	3100	0	0·000%
	Caoyan Village of Nanzheng County, Hanzhong City	1	50	0	2	3552	0	0·000%
Gansu (3)	Songshan Village of Tange Town in Wushang County, Tianshui City	1	50	0	1	2000	0	0·000%
	Hulinjia Town of Jishishan County, Linxia Hui Autonomous Region	1	50	0	1	2500	0	0·000%
	Xiqu Town of Minqin County, Wuwei City	1	50	0	1	2000	0	0·000%
Qinghai (6)	Shangluoma Village of Huangzhong County, Xining City	1	50	0	1	1080	0	0·000%
	Chengguan Town of Huzhu County	1	50	0	1	1180	0	0·000%
	Galeng Village of Xunhua County	2	110	3	1	876	0	0·000%
	Shazhuyu Village of Dulan County in Haixi State, Qinghai Province	3	150	3	1	900	0	0·000%
	Qingshizui Village of Menyuan County of Haibei State, Qinghai Province	1	100	2	1	587	0	0·000%
	Gushan Town of Minhe County, Qinghai Province	1	50	0	1	1230	0	0·000%
Ningxia (5)	Sanhe Town of Haiyuan County, Zhongwei City	1	50	0	3	7962	2	0·025%
	Wangtuan Town of Tongxin County, Wuzhong City	1	50	0	2	1900	0	0·000%
	Hongyao Town of Xiji County, Guyuan City	1	50	0	3	3730	0	0·000%
	Chengguan Town of Pingluo County, Shizuishan City	1	50	0	2	2727	0	0·000%
	Lijun Town of Yongning County, Yinchuan City	1	50	0	2	35571	0	0·000%
Guangxi (6)	Xiashi Town of Pingxiang, Chongzuo City	1	84	0	2	5950	0	0·000%
	Xiaorong Village of Rongshui Town of Rongshui County, Liuzhou City	1	68	0	3	3017	0	0·000%
	Qisha Town of Fangchenggang District, Fangchenggang City	1	79	0	4	12750	0	0·000%
	Zhongliang Town of Jinxiu County, Laibin City	1	100	1	2	4767	1	0·020%
	Der Town of Longlin County, Baise City	1	75	0	2	5770	0	0·000%
	Qibainong Village of Dahua County, Hechi City	1	81	0	2	2515	0	0·000%
Tibet (6)	Chengguan District, Lhasa City	1	50	0	1	5000	0	0·000%
	Linzhi County of Linzhi Area	5	350	0	1	3523	0	0·000%
	Sangyezhu District, Shigatse Prefecture	1	50	0	1	5320	0	0·000%
	Qiongjie County, Shannan Prefecture	4	200	0	—	—	—	—
	Anduo County, Nagqu Prefecture	4	200	0	4	1500	0	0·000%
	Ritu County and Gaji County, Ali Prefecture	4	210	2	—	—	—	—
TOTAL (83)		93	7,022	16	161	317,496	21	0·007%

### TT and CO in village residents in TRA

Within the 83 suspected trachoma-endemic areas, TRA for TT and CO was carried out in 161 villages in 14 provinces with 317,496 examined persons aged 15 years or older. In Tibet, there was no villages near two primary schools, thus the TRA for TT and CO cannot performed there. A total of 21 cases of confirmed TT, who were all 50 years old or above were found in 5 provinces (Inner Mongolia, Anhui, Guizhou, Ningxia, Guangxi). Only in 3 areas (Danshan Town and Ningnan County in Sichuan, and Baochang Town in Inner Mongolia), the prevalence of TT was above the threshold 0.2% (Table 2). Across all of the 161 villages, only 1 case of CO was found, the prevalence of CO is less than 0.1%.

### TT and CO cases in population-based by local ophthalmologists

After TRA, we mobilized local ophthalmologists to conduct population-based survey for TT in the provinces with TRA and pilot study. These local ophthalmologists got a training course about diagnosis of TT and population-based survey. Since there were no enough local ophthalmologists in Tibet and Guangxi to conduct this survey, so this survey was conducted in only 14 provinces. Table 2 showed the results. We found 1,296 TT cases in 87,540,342 persons aged 15 years or above in 55,481 villages. Although the prevalence of TT was above 0.2% in three TRA areas (Tables 1 and 2), the province-level prevalence of TT was <0.2% across all the 16 provinces (Table 3). The prevalence was unadjusted for age and gender.

**Table 3:** Assessment result for TT and CO in 14 provinces by local ophthalmologists.

Province	No. of villages Examined	No. of villagers Examined	No. of Villagers with TT	Prevalence of TT
Shandong	4514	3960930	0	0.000%
Sichuan	206	527692	55	0.010%
Hebei	40414	49616747	1	0.000%
Inner Mongolia	660	616234	42	0.007%
Liaoning	155	63073	0	0.000%
Anhui	540	1708899	74	0.004%
Henan	22	59830	37	0.062%
Hainan	2614	1875277	29	0.002%
Guizhou	23	41651	7	0.017%
Yunnan	122	1011414	34	0.003%
Shaanxi	5322	25750000	987	0.004%
Gansu	390	720045	25	0.003%
Qinghai	92	88550	0	0.000%
Ningxia	407	1500000	5	0.000%
TOTAL	55481	87540342	1296	0.001%

### Treatment of Identified trachoma cases

Therapeutic interventions for TF and TT patients in the 16 provinces were completed by December 2014. The 16 TF patients were cured with azithromycin. 1,334 TT cases (including 17 cases in pilot assessment, 21 cases in TRA, and 1296 cases in the population-based survey by local ophthalmologists) received successful treatment.

## Discussion

### Significance of assessing trachoma in mainland china

WHO document in 2012 stated that trachoma was still considered prevalent in 8 countries, among the 27 countries in the Western Pacific region, including China. Based on reports for trachoma control efforts and relevant statistics in China during recent decade, it was on the contrary understood that trachoma had been effectively controlled and eliminated as a cause of vision loss of public health importance. There was no record of trachoma or TT in the last decade in medical reports from county and provincial hospitals, despite a quite exhaustive health data reporting system. In the absence of an assessment based on WHO recommended methodologies, it was understandable that China was included among those countries suspected of having trachoma as a public health problem. Furthermore, a new trachoma survey was a good opportunity to introduce the standardized WHO Simplified Grading System, which was not commonly used in China, leading to inaccurate reporting of the disease against the internationally adopted criteria. A trachoma assessment would provide the basis upon which to determine if any further action was required to demonstrate that the goal of WHA Resolution 51.11 had been reached.

### Project design

China is a large geographic area with a huge population and substantial regional differences in socio-economic and hygienic conditions. A project of this scope to establish the public health priority that should be assigned to the elimination of trachoma under such a varied geographical and demographic environment was unprecedented.

Although TRA has never been validated for the assessment of elimination of trachoma as a public health problem, and because of the absence of any trachoma control intervention for many years, it was considered the most suitable approach to identify remaining endemic areas of trachoma. In each suspected trachoma-endemic area, one or more primary schools with the worst socio-economic conditions, worse water supply and poorest schooling conditions were therefore selected for TRA.

TRA findings were then used to determine whether a standard population-based survey was needed to confirm the existence and severity of trachoma. This two-step strategy was the key in the design of a feasible approach to establishing whether trachoma was still a public health issue in China. With a threshold of 4 cases of TF among 50 children examined in any school to start a population-based survey, none of the 97 suspected areas warranted such a survey.

### Results and future work

Our results show that nowhere near the location with the number of TF cases required for public health concern were found in any of the suspected areas. Although we found three areas (two areas in the pilot assessment, one areas in TRA), which belong in the remote and poor areas, with the prevalence of TT higher than 0.2%, after examining other areas nearby, there were fewer TT cases and the result didn’t reach our threshold to conduct the population-based survey for TT. After TRA, we mobilized local ophthalmologists to conduct population-based survey for TT in the provinces with TRA and pilot study, and found that the prevalence of TT in each surveyed province was not more than 0.2%. Moreover, trachoma was not a cause of visual impairment based on the results of national epidemiological surveys and rapid assessment for avoidable blindness. And eye care system at each level was able to manage existing and incident cases. So no further investigation was carried out, but more attention would still be given to these areas in the future work, especially the remote and poor villages. With negative historical record relevant to trachoma in the other 15 provinces of China and with socio-economic development, improvement of hygiene and GDP ranking better than in the 16 surveyed provinces, it may be concluded that trachoma is no longer a public health issue in China. There are some limitations in our paper, and the main one is the coverage area and population in the assessment. Based on our study design, we conduct TRA in which just those selected districts and people were examined. And the conclusion we reach is based on TRA rather than population-based survey. Despite the positive result from the assessment, proper screening and improvement in hygiene condition should still be maintained, and continuous monitoring on trachoma epidemic condition would also be conducted. Our methodology is generalizable among countries with large population, vast territory and great regional diversity which can use TRA for initial assessment and then population-based survey when needed.

There are several underlying reasons that support our findings of trachoma elimination in China. First, China has enjoyed rapid socio-economic development during the recent decades with significant improvement in income and living conditions, including quality of housing and water supply. From 1990 to 2014, population benefited from water improvement programs of varied types were increased by 37%, with the number of nearly 249 million persons. And 262 million households in rural areas were benefited from latrine improvement programs. Second, with an expanding public health system, the Chinese government has attached substantial importance to implementation of basic hygiene measures, which are also effective for the elimination of trachoma. Third, the centers for disease prevention and control, maternal and child care centers and health education organizations have been active at all levels in working with professional institutions in trachoma control. Fourth, there have been consistent efforts in trachoma control by the Chinese Society of Ophthalmology. Currently, ophthalmologists in China actively participate into a variety of activities for blindness prevention, including frequent screening for eye diseases, free treatment for TF patients by Azithromycin, health education and publicity at community level. Fifth, hygiene in rural environments has improved in conjunction with public works directed at drinking water and sanitation improvements. Sixth, the massive use of antibiotics is also likely to have curbed the transmission of Chlamydia. Last, but not least, results from 16 Rapid Assessments of Avoidable Blindness and recent epidemiological surveys also provided strong evidence that trachoma is not a significant cause of blindness [4, 5, 15, 16].

This large study suggested that trachoma was not a public health problem in 16 provinces that were previously suspected to be endemic. These findings will facilitate planning for elimination of trachoma from P. R. China.

## Supporting information

S1 Checklist (DOC)

S1 Table (DOCX)

S1 Document (PDF)

S2 Document (PDF)

S1 List of literatures (XLSX)
