# Correction: Assessment of trachoma in suspected endemic areas within 16 provinces in mainland China

**DOI:** 10.1371/journal.pntd.0007531

**Published:** 2019-06-24

**Authors:** Jialiang Zhao, Silvio Paolo Mariotti, Serge Resnikoff, Yuqin Wang, Shicheng Yu, Mingguang He, Yingchuan Fan, Haidong Zou, Wenfang Zhang, Yading Jia, Lihua Wang, Huaijin Guan, Xiao Xu, Leilei Zhan, Lei An, Quanfu Ye, Ningli Wang

The first author, Jialiang Zhao, is incorrectly noted as the corresponding author. The correct corresponding authors are Quanfu Ye and Ningli Wang. Dr. Ye's email address is: quanfu_ye@163.com. Dr. Wang’s email address is: wningli@163.com.
