# Supplementary material for: Assessment of trachoma in suspected endemic areas within 16 provinces in mainland China
Source: PLoS Negl Trop Dis. 2019 Jan 28;13(1):e0007130. doi: 10.1371/journal.pntd.0007130 (PMC6366720; doi:10.1371/journal.pntd.0007130)
Supplement: S1 Table — (DOCX) [file pntd.0007130.s002.docx]

S9 Water and latrines improvement situation in rural areas of China

**Table 1 Water improvement situation in rural areas of China**

| **Year** | **Population benefited**  **(10,000 persons)** | **Waterworks** | | **Hand pump well** | | **Rainwater collection** | | **Others** |
| --- | --- | --- | --- | --- | --- | --- | --- | --- |
|  |  | **No.** | **Accumulated beneficiary (10,000 persons)** | **No. (10,000)** | **Accumulated beneficiary (10,000 persons)** | **No.**  **(10,000 persons)** | **Accumulated beneficiary (10,000 persons)** | **Accumulated beneficiary (10,000 persons)** |
| **1990** | 66585.0 | 332044 | 27128.0 | 3311.0 | 17251.0 | / | / | 22206.0 |
| **2000** | 88112.2 | 674758 | 52669.5 | 4891.0 | 22264.8 | 1622886 | 1002.3 | 12175.6 |
| **2010** | 90833.9 | 629164 | 68158.5 | 6006.8 | 15172.8 | 2172278 | 1285.0 | 6217.5 |
| **2014** | 91511.3 | 555362 | 75470.9 | 9053.4 | 10022.4 | 2031063 | 1432.9 | 4585.1 |

**Table 2 Latrines improvement situation in rural areas of China**

| **Year** | **No. of households in rural areas (10,000)** | **Accumulated households with sanitary latrines (10,000)** | **Popularizing rate of sanitary latrines (%)** | **Popularizing rate of sanitary latrines for non-hazardous disposal of excreta (%)** |
| --- | --- | --- | --- | --- |
| **2005** | 24843.1 | 13740.1 | 55.3 | / |
| **2010** | 25415.4 | 17138.3 | 67.4 | 45.0 |
| **2014** | 26219.1 | 19939.3 | 76.1 | 55.2 |
