# Supplementary material for: Assessment of trachoma in suspected endemic areas within 16 provinces in mainland China
Source: PLoS Negl Trop Dis. 2019 Jan 28;13(1):e0007130. doi: 10.1371/journal.pntd.0007130 (PMC6366720; doi:10.1371/journal.pntd.0007130)
Supplement: S1 Document — (PDF) [file pntd.0007130.s003.pdf]

WORLD HEALTH ORGANIZATION
Prevention of Blindness & Deafness
Geneva, Switzerland

WHO/PBD/GET/00.6
Distr.: Limited
Original: English

FIRST NATIONAL WORKSHOP ON THE ASSESSMENT AND MANAGEMENT OF TRACHOMA IN THE PEOPLE'S REPUBLIC OF CHINA

Kunming, Yunnan Province
People's Republic of China

1-4 November 1999

Conclusions and Recommendations

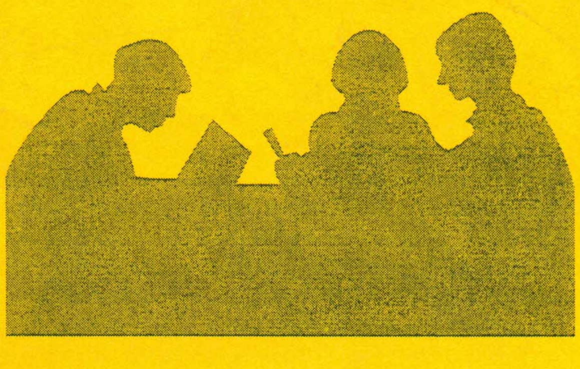

© World Health Organization. 2000

This document is not issued to the general public,
and all rights are reserved by the World Health Organization (WHO).
The document may not be reviewed, abstracted, quoted, reproduced
or translated, in part or in whole, without the prior written permission of WHO.
No part of this document may be stored in a retrieval system
or transmitted in any form or by any means - electronic, mechanical
or other - without the prior written permission of WHO.
The views expressed in documents by named authors
are solely the responsibility of those authors.

PREAMBLE

This workshop is a follow-up to the recommendations made on the occasion of the WHO/Ministry of Health/International NGDO Coordination meeting for the Prevention of Blindness in China, held in Beijing in March 1998 (WHO/PBL/98.69). During this meeting, available information on trachoma was reviewed, showing that foci of blinding trachoma still persist in different provinces of China and that active trachoma can be found in some counties in the 12 following provinces, i.e., Anhui, Beijing, Gansu, Guangxi, Henan, Jiangsu, Jilin, Liaoning, Shangdong, Shanxi, Tianjin and Yunnan.

Representatives of 13 provinces attended the workshop (see Annex 2 for list of participants). Nine of these provinces were included in the above-mentioned selection. Others such as Chong Qing, Quing Hai, Shaanxi and Ningxia Hui Autonomous Region were also invited and represented as they are suspected to have trachoma.

The workshop which was organized jointly between WHO/PBD and the Beijing Institute of Ophthalmology, with support received from the WHO Alliance for the Global Elimination of Trachoma, was hosted by the health authorities of the Yunnan Province. Mr Du Kelin, Deputy Director of the Department of Health of the Yunnan Province opened the workshop.

The specific objectives of this workshop were (1) to promote the SAFE strategy (Surgery, Antibiotics, Facial cleanliness and Environmental changes), (2) to bring the participants up-to-date on the current status of trachoma in endemic countries, (3) to review activities presently undertaken by NGOs in endemic provinces, (4) to investigate future potential partnerships, and (5) to train the participants in the development of appropriate plans of action and in the identification of suitable strategies.

All the objectives were reached and presentations were made on all the items listed in the agenda (Annex 1) followed by general discussions. Four working groups were formed on the third day for training in the development of plans of action and the identification of suitable strategies. Trachoma information was presented by a number of participating provinces and is reported in Annex 3 of this report.

A field visit, organized on the last day of the workshop allowed the participants to take part in a demonstration of trachoma screening among school children using the WHO Simplified Grading System.

Conclusions and Recommendations

1. Declaration of support for the Global Initiative for the Elimination of Avoidable Blindness (Vision 2020)

The Minister of Public Health of the People's Republic of China signed the declaration of support for the Global Initiative for the Elimination of Avoidable Blindness (Vision 2020 – The Right to Sight) in September 1999. As the Global Elimination of Trachoma is an integral part of this Initiative, it is recommended that trachoma control be implemented nation-wide without delay.

2. Data collection and reporting

As part of the above-mentioned Initiative, it is recommended that trachoma data be reported based on the WHO Simplified Trachoma Grading System. Standardized data collection and reporting are essential for prioritization and planning. As reliable data on trachoma are currently unavailable for most provinces, it was recommended that data collection systems be institutionalized at both national and provincial levels.

3. Trachoma documentation

The group recommended that all WHO/PBD documentation on trachoma control be made available in Chinese for use at the provincial and county levels.

4. Training

It was recommended that the WHO Collaborating Centre in Beijing collaborate with the Chinese Ophthalmological Society for implementation of the WHO Simplified Grading System nation-wide and that further workshops be organized to train trainers in the use of that system.

5. The SAFE strategy (Surgery, Antibiotics, Facial Cleanliness, Environmental improvements)

The SAFE strategy was extensively discussed and the participants endorsed its principles. However, the strategy still requires to be formally adopted at the national level before implementation within provincial and county programmes.

6. Trichiasis surgery

In China, an estimated 6 million people require trichiasis surgery. As this is becoming a pressing priority, it is recommended that standardized training for trichiasis surgery be implemented.

7. Health education

It is recommended that the WHO Collaborating Centre in Beijing develop health education material and books consistent with the procedures of the SAFE strategy and disseminate them throughout the endemic provinces and counties.

8. Role of mid-level health personnel

During the discussions concerning the implementation of the SAFE strategy, the potential role of mid-level health personnel was discussed extensively. It was recommended that further considerations be given their potential involvement.

9. Partnerships

The Global Initiative for the Elimination of Avoidable Blindness (Vision 2020 – The Right to Sight), is a partnership between WHO, governmental and nongovernmental organisations (NGOs). The Group recognized the value of this partnership, in particular in terms of opportunities for International Nongovernmental and Developmental Organizations (INGDOs) to assist in the implementation of trachoma control programmes. It was recommended that these partnerships be further developed to include the development of model and demonstration projects, and that activities of INGDOs be coordinated at the national level.

10. Programme review

It was recommended that progress on trachoma control activities be reviewed regularly through national workshops including representatives of : (1) the Ministry of Public Health, (2) the National Committee for the Prevention of Blindness, (3) the provincial health authorities, (4) the PBL programmes, (5) the Chinese Ophthalmological Society, INGDOs and WHO. It was suggested that the next meeting be held within 12 months from the present workshop.

ANNEX 1

FIRST NATIONAL WORKSHOP ON THE ASSESSMENT AND MANAGEMENT OF TRACHOMA IN THE PEOPLE'S REPUBLIC OF CHINA

Kunming, Yunnan Province, China (1-4 November 1999)

AGENDA

DAY	AGENDA ITEM	SPEAKER
Monday 1 November	Opening ceremony<br>Election of Officers<br>Introduction of participants<br>Administrative announcements<br>Adoption of agenda	
	1. Epidemiology of trachoma	*Dr A.-D. Négrel, WHO*
	2. WHO Alliance for the Global Elimination of Trachoma	*Dr S. P. Mariotti, WHO*
	3. Trachoma in China : past and present	*Dr Sun Bao Chen, Beijing Institute of Ophthalmology*
	4. Presentation of Provinces (3)	*Province Representatives*
	5. Trachoma control and primary health care	*Dr K. Konyama, Jutendo University, Tokyo*
	6. Trachoma control integrated approach (SAFE strategy)	*Dr S. P. Mariotti, WHO*
	6.1 Surgery	*Prof. H. Taylor, University of Melbourne & WHO*
	6.2 Antibiotic treatment	*Dr A.-D. Négrel*
	6.3 Facial Cleanliness	*Prof. H. Taylor*
	6.4 Environmental changes	*Dr S. P. Mariotti*

DAY	AGENDA ITEM	SPEAKER
**Tuesday 2 November**	7. Information Education & Communication (IEC)	*Dr K. Konyama*
	8. Presentation of Provinces (3)	*Province Representatives*
	9. Presentation of Christoffel-Blindenmission (CBM)	*Dr A. Pyott, CBM/Cambodia*
	10. Trachoma simplified grading system	*Prof. H. Taylor*
	11. Basic principles of trachoma control management	*Dr A. D. Négrel*
	12. Presentation of Provinces (3)	*Province Representatives*
	13. Trachoma Rapid Assessment	*Dr A.-D. Négrel*
	14. Presentation of Provinces (3)	*Province Representatives*
**Wednesday 3 November**	15. Working groups to develop provincial priorities and plans of action (4 working groups)	*3 groups of Province Representatives*
	16. Presentation of provincial priorities and plans of action and discussion	***Facilitators:***<br>*- Dr K. Konyama*<br>*- Dr Sun Bao Chen*<br>*- Prof. Hugh Taylor*
	General discussion	
	Conclusions and Recommendations	
**Thursday 4 November**	Field Visit	*GROUP*

ANNEX 2

FIRST NATIONAL WORKSHOP ON THE ASSESSMENT AND MANAGEMENT OF TRACHOMA IN THE PEOPLE'S REPUBLIC OF CHINA

Kunming, Yunnan Province, China (1-4 November 1999)

LIST OF PARTICIPANTS

REPRESENTATIVES OF PARTICIPATING PROVINCES/COUNTIES

ANHUI PROVINCE

Dr Zhang Guo mei, Deputy Chief of Eye Department, Anhui Provincial Hospital, 17 Lujiang Road, Hefei, Anhui Province, People's Republic of China
(Tel. +86 551 265 2797 – Ext. 3279 (home); Fax. + 86 551 267 0607)

BEIJING PROVINCE

Professor Sun Bao Chen (see Collaborating Centre section)

CHONG QING PROVINCE

Dr Hong Li, Director of Eye Department, Third People's Provincial Hospital, No.104 Yu chong Qu pi ban shan cheng yie, Chong Qing Province, People's Republic of China
(Tel. +86 23 6351 4181 and 6351 5266)

HAINAN PROVINCE

Dr Liu Haisheng, Director of Eye Department, Hainan Provincial Hospital, Haikou 570311, Hainan Province, People's Republic of China
(Tel. +86 898 7866 4589; Fax. +86 898 7866 3485; E.mail. hsliu@hg.cninfo.net)

QING HAI PROVINCE

Dr Tian Kewu, Director of Eye Department, Qing Hai Provincial Hospital, 2 Gonghe Road, Xining, Qing Hai Province 810007, People's Republic of China *(Tel. +86 971 812 8359)*

LIAONING PROVINCE

Professor Zhang Bai-xin, Director of Eye Department, Liaoning Provincial Hospital, Wenyi Road 33, Shenhe District, Shenyang City, Liaoning Province, People's Republic of China
(Tel. +86 24 2414 7900 Ext. 8523)

NINGXIA HUI AUTONOMOUS REGION

Dr Yang Qiaoling, Director of Eye Department, Ningxia Provincial Hospital, Huhi Yuan Road Yin Chuan, Ningxia Hui Autonomous Region, People's Republic of China *(Tel. +86 951 208 3100)*

SHAANXI PROVINCE

Professor Ren Baichao, Director of Eye Department, Second Clinical Medical College, Xian Medical University Head of the Shaanxi Provincial Prophylactic Blindness Office, and Secretary of the Shaanxi Provincial Ophthalmology Association, No. 36 West Wu Road, Xian, 710004 Shaanxi Province, People's Republic of China *(Tel. +86 29 726 9963 (Hosp.); +86 29 727 6936 -Ext.29449)*

SHANDONG PROVINCE

Mr Zhou Wei, Vice-Director, Medical Division of Shandong Public Health Bureau, Shandong Province, People's Republic of China *(Tel. +86 531 295 3269)*

Professor Li Chunhua, Vice-Director, Shangdong Province Blindness Office, Langmao Mountain, Jinan, Shandong Province, People's Republic of China *(Tel. +86 531 297 7749)*

SHANXI PROVINCE

Dr Shi Zhengqi, Vice Director of Specialized Group for Prevention of Blindness in Shanxi, and Vice-Director of Shanxi Provincial Hospital, 252 Fu Dong Road, Taiyuan, Shanxi 030002, People's Republic of China *(Tel. +86 351 418 5441)*

Wu Zhili, Ophthalmic Nurse and Secretary of the Specialized Group for Prevention of Blindness in Shanxi, Shanxi Provincial Hospital, 252 Fu Dong Road, Taiyuan, Shanxi 030002, People's Republic of China *(Tel. +86 351 202 8215 Ext. 6528)*

SICHUAN PROVINCE

Dr Zhang Xiao Qi, Director, Sichuan Provincial Committee for Prevention of Blindness, Vice-Director, Eye Department, Sichuan Provincial Hospital, Qing Yang gong, Cheng du, Sichuan Province, People's Republic of China *(Tel. +86 28 776 9981 – Ext. 4467; Fax. +86 28 776 1935)*

Dr Zhou Jun Hua, Director of Eye Department, Sichuan Provincial Hospital, Qing Yang gong, Cheng du, Sichuan Province, People's Republic of China
(Tel. +86 28 776 9981 – Ext. 4467; Fax. +86 28 776 1935)

TIANJIN PROVINCE

Dr Sun Huimin, Director, Tianjin Medical University Eye Centre, No. 22 Qixiang Tai Road, 300070 Tianjin, Tianjin Province, People's Republic of China
(Tel. +86 22 2334 6430; Fax. +86 22 2334 6434)

YUNNAN PROVINCE

Professor Yuan Tianguo, Director of Eye Department, First People's Hospital, 172 Jinbi Road, Kunming 650032, Yunnan, People's Republic of China *(Tel. +86 871 363 4031 – Ext. 2252)*

Professor Jiang Chunguang, Director of Eye Department, The Red Cross Hospital of Yunnan, Qingnian Road, Kunming, Yunnan Province 650021, People's Republic of China
(Tel. +86 871 515 6650 Ext. 2880; Fax. +86 871 515 7157; E.mail John-Jiang@km169.net*)*

Dr Xiao Yungao, Vice Director of Eye Unit, The Red Cross Hospital of Yunnan, Qingnian Road, Kunming, Yunnan Province 650021, People's Republic of China
(Tel. +86 871 818 8841 (home); Fax. +86 871 515 6157)

Professor (Ms) Li Hanyu, Director of Eye Department, First Affiliated Hospital of Kunming Medical College, No.153 Xichang Road, Kunming, Yunnan Province, People's Republic of China
(Tel. (86) 8771 532 4888 (Ext.2712)

Prof. Lan Bingyan, President)
Prof. Lui Qiaobao, Vice-President) First People's Hospital
Prof. Zhang Shiyan, Vice-President) of Yunnan Province
Prof. Huang Yaozhong, Director of Education Department) Kunming
Dr Liang Zhisong, Vice-Director, Education Department)
Mr Han Bing, Education Department)

Dr Li Youda, Director, Yunnan Provincial Office of Prevention of Blindness
Mr Du Kelin, Vice-President, Yunnan Public Health Bureau
Mr Duan Qixiong, Vice-Director, Yunnan Public Health Bureau
Mr Liu Ronghua, Cadre, Yunnan Public Health Bureau

Representatives of WHO Collaborating Centres in the Western Pacific Region

Dr K. Konyama, Department of Ophthalmology, Juntendo University School of Medicine, 3-1-3 Hongo Bunkyo-ku,Tokyo, Japan *(Fax. 81338170260; Tel. 81338133111; E.mail:* juntenop@iris.dti.ne.jp & kkon@interlink.or.jp *(Temporary Adviser)*

Dr K. Ono, Ophthalmologist, Juntendo University Hospital, Department of Ophthalmology, 21-1-1 Hongo, Bunkyo-ku, Tokyo, Japan (*Tel. +81 3 3811 3111)*

Professor Sun Bao-Chen, Beijing Institute of Ophthalmology, 17 Hou Gou Lane, Chong Nei Street,Beijing 100730,People's Republic of China *(Fax.+86 10 6512 5617; Tel. + Fax. +86 106513 0796; E.mail. sunbaoch@public.bta.net.ch)*

Professor Hugh Taylor, Department of Ophthalmology,The University of Melbourne,The Royal Victorian Eye and Ear Hospital, 32 Gisborne Street,East Melbourne, Victoria 3002, Australia
(Fax. 61 3 9662 3859; Tel. 61 3 9929 8368; E.mail: htaylor@iris.medoph.unimelb.edu.au; & judy@iris.medoph.unimelb.edu.au) *(Temporary Adviser)*

Representatives of Nongovernmental Organizations

Dr Hanheng Liu, Representative of Helen Keller Worldwide, Director, Department of Ophthalmology, Hainan Provincial Hospital, Haikou, Hainan Province, People's Republic of China *(E.mail:* hsliu@hq.cninfo.net)

Dr Andrew Pyott, Representative of Christoffel-Blindenmission, P.O. Box 632, House 24A, Street 322, Boeung Keng Kong, Chalmar Morn District, Phnom Penh, Cambodia (*E.mail*: apyott@forum.org.kh)

WHO Secretariat

Dr S. P. Mariotti, Ophthalmologist, Prevention of Blindness and Deafness (PBD), World Health Organization Avenue Appia, CH-1211 Geneva, Switzerland *(Fax. 41 22 791 4772, Tel. 41 22 791 3491, E.mail:* mariottis@who.ch)

Dr A.-D. Négrel, Ophthalmologist, Prevention of Blindness and Deafness (PBD), World Health Organization Avenue Appia, CH-1211 Geneva, Switzerland *(Fax. 41 22 791 4772, Tel. 41 22 791 2652, E.mail:* negrela@who.ch)

ANNEX 3

AVAILABLE INFORMATION ON TRACHOMA PRESENTED DURING THE WORKSHOP

Anhui Province 12

Hainan Province 13

Liaoning Province 14

Ningxia Hui Autonomous Region 15

Shaanxi Province 16

Shandong Province 17

Shanxi Province 18

Sichuan Province 20

Zhaotong County, Yunnan Province 22

ANHUI PROVINCE

1. Background Information*

Population:	59 million (Urban: 15.3 million)
Area:	130 000 km^2
Physical features:	Mainly plains and hills
Administrative divisions:	16 cities and 65 counties
Capital:	Hefei

2. Prevalence of trachoma

The prevalence of trachoma is estimated to be 12.5% varying between 19.5% in the most prevalent areas and 4.5% in the least prevalent areas.

In 1990, an epidemiological survey on trachoma in Fuyang revealed a prevalence of 10.78% in the 12 to 15 year-old population group. The survey in Langxi indicated that the prevalence in the 6 to 10 year-old population group was 17.38%. The findings of these two surveys show that active trachoma is a major problem in certain parts of the Anhui Province.

In Huaibei, the prevalence of trachoma is high in poor areas and where water is scarce. In the mountainous areas of Western Anhui, consisting of some of the poorer areas in the province, there are repeated infections of trachoma. Trachoma patients cannot afford treatment and environmental sanitation is poor. Despite the large number of TT cases requiring trichiasis surgery and the high demand from the communities for such an intervention, four major obstacles prevail, i.e.:

(i) the village primary eye care workers cannot perform surgery;
(ii) there are no means of transportation;
(iii) patients cannot afford surgery;
(iv) unavailability of outreach services and absence of referral system.

3. Trachoma control activities

Although trachoma is prevalent in this province, it is not considered as a priority by the provincial health authorities. No past or present control activities have been reported.

4. Plans for the future

(1) Undertaking of a trachoma epidemiological survey
(2) Reformulation of the provincial trachoma control programme
(3) Implementation of the SAFE strategy
(4) Integration of trachoma control within village-level primary health care

* Reference: www.cbw.com/general/gintro.htm

HAINAN PROVINCE

1. Background information*

Population: 7.01 million (Urban: 1.8 million)
Area: 34 000 km^2
Physical features: Second largest island in China 1/3 mountainous
Administrative divisions: 3 cities, 9 counties, and 7 autonomous counties
Capital: Haikou

2. Prevalence of trachoma

In the 1950s, the prevalence of trachoma in Hainan Province was close to the average national level, i.e., approximately 50%. In August 1999, the Hainan Provincial Hospital, in collaboration with the Zhongshan Ophthalmology Center and Helen Keller Worldwide, conducted a trachoma rapid assessment in Dongfang and Changjiang counties which provided the following results:

County	Samples	Active trachoma (%)	TT (%)	TS (%)
Dongfang	1292	1.4	1.3	7.5
Changjiang	1135	2.3	0.54	7.4

3. Trachoma control activities

No past or present activities were reported during this workshop.

4. Plans for the future

Although the prevalence of active trachoma in both counties is relatively low, that of TS is fairly high which indicates that TT is bound to increase further in the coming years. Collaboration to tackle the uptake of trichiasis surgery should be sought in order to reach the goal for Global Elimination of Blinding Trachoma by the year 2020.

* Reference: www.cbw.com/general/gintro.htm

LIAONING PROVINCE

1. Background information*

Population:	40.4 million (Urban: 25.7 million)
Area:	150 000 km^2
Physical features:	High in the west and east, low plains in the central and southern coastal areas
Administrative divisions:	19 cities, 34 counties, and 5 autonomous counties
Capital:	Shenyang

2. Prevalence of trachoma

Before the 1960s, trachoma was one of the main causes of blindness in the Province. Along with the economic development, many efforts were made to control trachoma. At present trachoma is the third or fourth cause of blindness in the Province.

A survey on blindness and low vision conducted in the 1980s indicated a blindness prevalence rate of 0.43%. The causes were, in order of priority: cataract, glaucoma and trachoma-induced corneal diseases. A trachoma epidemiological survey carried out in the Benxi region has revealed a prevalence of around 12%.

3. Trachoma control activities

The following activities are planned to be implemented in the near future:

1. Integration of trachoma control activities into PHC during the course of 2000;

2. Training of trachoma control personnel in collaboration with CBM : The establishment of a five-year training programme for eye care personnel at the county, township and village levels is planned using the county hospitals as a base for training. The expected outcome for training is 200 ophthalmologists at county level, 500 ophthalmologists at township level and 2000 health workers at village level.

3. Organization of Information, Education and Communication (IEC)campaigns in collaboration with the SightFirst/China Action Plan (including the integration of PEC in school health education);

4. Provision of surgical instruments to township hospitals for trichiasis surgery.

4. Plans for the future

1. To apply the SAFE strategy as recommended by both WHO and the National Blindness Prevention Programme to reach the goal of Global Elimination of Blinding Trachoma by the year 2020 (GET 2020).

2. To develop a trachoma control plan for the Province in the framework of the SightFirst/China Action Plan.

* Reference: www.cbw.com/general/gintro.htm

NINGXIA HUI AUTONOMOUS REGION

1. Background information*

Population:	5.0 million (Urban: 1.7 million)
Area:	66 000 km^2
Physical features:	Plateaux in the South and East, plains in the North, mountains in Southwest and along Western border
Administrative divisions:	4 cities and 16 counties
Capital:	Yinchuan

2. Prevalence of trachoma

In the 1950s, trachoma was the first cause of blindness in that region with a very high prevalence rate. Today, it is no longer considered as the main cause of blindness although trachoma prevalence is estimated to be about 10% and the percentage of cases leading to trichiasis is estimated to be about 0.1%.

3. Trachoma control activities

No trachoma control programme exists in the Province. However, trachoma treatment is carried out in hospitals at the township, county and provincial levels.

4. Plans for the future

Not reported during this workshop.

* Reference: www.cbw.com/general/gintro.htm

SHAANXI PROVINCE

1. Background information*

Population:	34.4 million (Urban: 12 million)
Area:	190 000 km^2
Physical features:	Plateaux in the North, plains in the central areas and mountains in the South
Administrative divisions:	8 cities and 89 counties
Capital:	Xi'an

2. Prevalence of trachoma

The last survey on eye diseases conducted in 1987 showed that in certain counties and townships of the mountainous areas of the Province, the prevalence of trachoma was estimated to be as high as 20%, especially in poor areas such as the prefectures of Yulin, Shangluo, Hanzhong and Yanan.

In poor areas where water is scarce, the prevalence rates were estimated to be 10% for TF and TI, 5% for TT and 2% for CO. In major and medium-sized cities of developed areas, the prevalence of trachoma was estimated to be approximately 5%, of which 1% were TT cases.

3. Trachoma control activities

In poor areas, trichiasis surgery is performed at the county hospital level by ophthalmologists only. As township health centres and village clinics have difficulties in diagnosing trachoma, trichiasis surgery is not performed at that level. Consequently, many patients are reported to be plucking their eyelashes although there exist no statistics to confirm that this is the case.

The majority of households, particularly in poor areas, share one towel and one basin for face-washing. Children rarely wash their faces and latrines are basic.

4. Plans for the future

1. Implementation of a trachoma or eye disease epidemiological survey pending financial support and collaboration from WHO and CBM;

2. Training of eye care personnel (medical doctors from township health centres and village health workers) for diagnosis of trachoma and trichiasis surgery.

* Reference: www.cbw.com/general/gintro.htm

SHANDONG PROVINCE

1. Background information*

Population: 86.4 million (Urban: 44.5 million)
Area: 150 000 km^2
Physical features: In lower Yellow River Valley, hills in central region and on the Eastern peninsula, plains in the north, west and central east, narrow lowlands in the South and along the South-Eastern coast
Administrative divisions: 25 cities and 86 counties
Capital: Jinan

2. Prevalence of trachoma

In the early days following the founding of the People's Republic of China, trachoma was considered to be the leading cause of blindness.

In 1987, a provincial survey on blindness prevalence carried out over the entire province (and based on the national standards set in 1979) indicated that this was still the case. The major eye diseases found in this survey were (in order of priority): trachoma, ametropia/low vision, senile cataract, corneal diseases and, disorders of the ocular fundus. The prevalence of trachoma indicated 15.31% of which 10.69 % was active trachoma and 4.62% active trachoma with scar formation with mild and moderate infections representing 69.94% and 30.06% each. These figures were found to be relatively lower in the ≤ 10 year-old age group but increased significantly with age.

A trachoma survey carried out in 1992 in Pingyin County of the Province (using the WHO's simplified grading system) showed a lower prevalence of active trachoma (9.6%) than that revealed in 1987. The prevalence of children under 10 years old was found to be 3.7% but increased with age.

The findings also showed that prevalence of low vision/blindness due to trachoma (CO) was was only 0.29% and that the prevalence of trichiasis surgery represented 0.55% indicating that trachoma is no longer a major cause of blindness. Moderate cases of trachoma account for only a minority of cases and severe cases are rarely seen.

3. Trachoma control activities

Prevention

During the 1950s and 1960s, a number of large-scale campaigns for trachoma screening and treatment, and for education on improved hygiene have been conducted by the Provincial Bureau for Trachoma Control. During the 1980s and up to the mid-90s, blindness prevention programmes were established including trachoma control. Since, blindness prevention including trachoma control has been integrated into primary health care. Major activities comprise health education and promotion of healthy behaviours and habits.

* Reference: www.cbw.com/general/gintro.htm

Treatment

Outpatient trachoma cases with visible symptoms are treated with topical preparations, including rifampin, chloramphenical and tetracycline, usually in the form of eye drops or eye ointment.

The treatment of complications, particularly entropion-trichiasis, corneal pannus and corneal opacity, involves mainly electrolysis for trichiasis and several other procedures for the correction of entropion.

4. Plans (recommendations) for the future

1. **Health education**: In order to intensify the effectiveness of health education and accelerate healthy behaviours and habits, further efforts are necessary, with increased input, in-depth research and innovative methods.

2. **Environment**: In rural, mountainous and economically underdeveloped areas, environmental improvement remains a priority.

3. **Treatment**: Attention should be given to the development of effective drugs and to the prevention of complications.

4. **Trachoma surveys** should be conducted in economically underdeveloped areas in order to determine strategic measures for effective control.

SHANXI PROVINCE

1. Background information*

Population:	30.1 million (Urban: 15.1 million)
Area:	150 000 km^2
Physical features:	Plateau and Mountains in the Eastern part. Lowlands, valleys and basins in the central part
Administrative divisions:	10 cities and 96 counties
Capital:	Taiyuan

2. Prevalence of trachoma

Two epidemiological surveys on eye disease conducted in 1986 and 1987 in the Shanxi Province and involving 100 000 urban and rural inhabitants have showed a blindness rate of 0.318%. Out of a population of 30 million, this equals to a total number of 95000 blind of which 5.8% are trachoma blind. As there has been no further survey since, data on trachoma prevalence is incomplete.

According to the statistics derived from the Shanxi Provincial Eye Hospital in 1998 and which concerned 145 000 patients, trachoma patients (including 2256 cases with complications) accounted for 1.56% of all outpatient cases.

3. Trachoma control activities

Although trachoma is prevalent in that province, no past or present trachoma control activities have been reported during this workshop.

4. Plans for the future

No plans were presented during this workshop.

* Reference: www.cbw.com/general/gintro.htm

SICHUAN PROVINCE

1. Background information*

Population:	111 million (Urban: 28.2 million)
Area:	560 000 km^2
Physical features:	Basin in the East, plateau in the West, mountain in the Central South, highland swamps in the Central North.
Administrative divisions:	3 autonomous prefectures, 19 cities,165 counties, 8 autonomous counties, and 1 industrial-agricultural district
Capital:	Chengdu

2. Prevalence of trachoma

During the 1950s, trachoma was the main cause of blindness in the Province. Following three decades of trachoma efforts and the increase in the standard of living and education, trachoma has dropped from first to second cause of blindness. A 1998 review of a survey on blindness and low vision conducted in the early 80s revealed a prevalence of blindness of 0.4% (cataract: 42.86% and trachoma: 18.97%) and a prevalence of low vision of 0.67% (cataract: 44.83% and trachoma: 15.38%). Data obtained for specific areas are presented as follows:

Year	Area	Samples	Trachoma cases	Prevalence (%)	Blepharelosis
1981	Naxi (Southern Sichuan)	3216	1128	35.08	0.81
1982	Dachuan (Northern Sichuan)	21869	12876	58.88	1.00
1983	Zhaojue (Western Sichuan)	4048	1338	35.05	0.16
1983	Ningnan (Western Sichuan)	5413	3768	69.61	2.86
1984	Dianjiang (Eastern Sichuan)	4690	1352	28.83	0.19
1984	Fuling (Eastern Sichuan)	4378	813	18.57	0.09

3. Trachoma control activities

- Integration of PEC into PHC at the programme level since 1990.
- Collaboration with CBM in training of eye care personnel at county, township and village levels :

- County level:	170 ophthalmologists & 120 nurses
- Township level:	700 ophthalmologists
- Village level:	700 eye care workers

- Dissemination, through PHC, of information on the control of eye diseases.
- Integration of PEC education in school health education.

* Reference: www.cbw.com/general/gintro.htm

- Provision of instruments for the correction of blepharelosis to 200 ophthalmologists at the township level.

4. Plans for the future

1. To respond proactively to the commitment made by the Ministry of Health and the National Office of Blindness Prevention to WHO to reach the goal of global elimination of blinding trachoma by the year 2020.

2. To establish a trachoma control programme in the Province.

3. To seek support from and collaboration with the provincial government as well as WHO and other international organizations to implement the SAFE strategy.

4. To implement trachoma rapid assessments in areas with a high prevalence of trachoma, subject to the assistance and cooperation of WHO and CBM.

ZHAOTONG COUNTY, YUNNAN PROVINCE

1. Background information*

Zhaotong County is a poor and remote area of the Yunnan Province with a population of 700 000 consisting mainly of ethnic minority groups. The county counts 30 townships and 300 villages.

A primary health care network exists and the health personnel consists of 10 ophthalmologists, 5 nurses, 15 ENT doctors and 15 part-time general practitioners.

2. Prevalence of trachoma

A county-wide epidemiological survey on blindness completed in 1998 showed a prevalence of blindness of 0.43%. Among the results, trachoma was listed as the third cause of blindness after cataract and corneal opacities with a prevalence of 17%, broken down according to the level of severity as follows:

WHO trachoma grading	Population	Prevalence
TF	21000	3%
TI	21000	3%
TS	30800	4.4%
TT	66500	9.5%
CO	700	0.1%

3. Trachoma control activities

Although trachoma is considered as a public health problem in the Zhaotong County, no past or present trachoma control activities have been reported during this workshop.

4. Plans for the future

A plan of action for trachoma control was prepared and presented to the group on the last day of the meeting (see Annex 4).

* Reference: www.cbw.com/general/gintro.htm

SUMMARY OF AVAILABLE INFORMATION ON TRACHOMA PER PROVINCE

Province	*Population (M)*	*Prevalence of Blindness (%)*	*Major causes of blindness*	*Number of ophthalmologists*	*Number of Cataract per Year*	*TF/TI (%)*	*TT (%)*	*Number of trichiasis surgery per year*	*Major PBL activities*	*NGOs*
**Anhui**	59	0.39	Cataract<br>Glaucoma<br>Trachoma	1000	11000	10.2	11.5	2000	HE, CS, PEC&MLP, environmental change, TTS	CBM
**Chong Qing**	30	0.45	Cataract<br>Glaucoma	600	5600	4.5	1.2	150	HC, HE, PH	-
**Hainan**	7	0.7	Cataract<br>Glaucoma<br>Corneal Opacities	83	1500	2	1	40	RA trachoma	HKI<br>SF
**Liaoning**	40	0.43	Cataract<br>Glaucoma<br>Corneal Diseases<br>Trachoma	1300	10000	5	0.5	1000	-	SF
**Ning Xia**	5	0.5	Cataract<br>Ocular Injury<br>Glaucoma	70	1000	5	0.5	80	Survey, MT,CH	-
**Qieng Hai**	5.3	0.43	Cataract<br>Corneal Diseases<br>Diabetic Retinopathy	88	1200	NA	NA	148	Information campaigns & training	CBM
**Shaanxi**	34	0.48	Cataract<br>Corneal Diseases<br>Posterior-pole Diseases	1000	14800	5	16	2000	CS & TTS	-
**Shanxi**	30	0.32	Cataract<br>Glaucoma<br>Corneal Diseases	650	7500	5	1	500	-	-
**Shandong**	86	0.34	Cataract<br>Corneal Opacities<br>Trachoma	1600	18000	9.6	0.84	2000	-	-
**Sichuan**	111	0.43	Cataract<br>Trachoma	1030	15000	20	1.2	2000	PHC, PEC, CS, TTS	CBM
**Tianjin**	9	0.3	Cataract<br>Glaucoma<br>Diabetic Retinopathy	400	10000	4	0.4	30	TTS, survey, MT, CH	CBM
**Yunnan**	40	0.57	Cataract<br>Corneal Opacities<br>Trachoma	1000	7800	6.7	1.1	10000	PEC, PBL, ACPHC	SF<br>CBM<br>MED

HE: Health Education
PEC: Primary Eye Care
PBL: Prevention of Blindness
TTS: Trichiasis Surgery
CS: Cataract Surgery
RA: Rapid Assessment

PHC: Primary Health Care
MT: Mobile Teams
MLP: Mid-level personnel
ACPEC: Development of Advanced County PHC
HC, CH : Health Centre, County Hospital
& PH & Province Hospital referral

CBM: Christoffel-Blindenmision
HKI: Helen Keller International
MED: Mekong Eye Doctors
SF: SightFirst China Action Plan

ANNEX 4

PROPOSED PLAN OF ACTION FOR TRACHOMA CONTROL IN ZHAOTONG COUNTY

GOAL: Elimination of (blinding) trachoma by the year 2015

TASKS: (1) Provide appropriate treatment to 72 800 trachoma cases (TF, TI)
(2) Perform surgery on 66 500 trichiasis cases with tarsal rotation procedure

I. BACKGROUND

The results of the epidemiological survey on blindness completed in 1998 and reported earlier, have revealed on the one hand (low prevalence of TF & TI) the positive effects of factors such as the availability of medical services, environmental and sanitation improvement, and on the other hand (high prevalence of TT), the existing deficit in terms of uptake of trichiasis surgery.

It is planned that, with the support received from the county, township, and village governments, trachoma control activities will be initiated by the Blindness Prevention Bureau. Primary eye care will be integrated into the existing PHC networks which will be consolidated.

II. STRATEGY

It is proposed to implement the SAFE strategy according to the following pattern:

S (Surgery): For treatment of trichiasis cases (TT)

Where? At village and township levels

By whom? Primary eye care workers, general practitioners, ophthalmologists

Procedure? Tarsal rotation

Cases? Trichiasis cases identified during the epidemiological survey will be referred to the village clinic for surgery

A (Antibiotics): For treatment of active trachoma (TF and TI)

Where? At village and township levels

By whom? Primary eye care workers at the village level for distribution of antibiotics and supervision of treatment

Treatment? 1% tetracycline eye ointment twice daily for six weeks

F (Face washing): Promotion of the use of clean water and individual towels

In collaboration with the Village Patriotic Health Team and Schools, primary eye care workers will play a role in information, education and communication particularly concerning children.

E (Environmental Change) :

With the participation of the entire community and supervision by PEC workers, work will be organized by the township and village commissions for the improvement of water supply and sanitation and for the promotion of healthy behaviours in villages and towns.

III. TRAINING OF PERSONNEL

Objectives: To enable PEC workers to :

1. use the WHO trachoma grading system for screening and diagnosis of trachoma,
2. disseminate trachoma control information (IEC),
3. distribute and supervise treatment,
4. perform trichiasis surgery.

Duration: 6-month training including 2 to 3 rounds of one week each per township.

Site: Township health centre

Trainers: County and township level ophthalmologists

Participants: 100

IV. PILOT STUDY

Three townships corresponding to the criteria of high prevalence of TT, and lack of infrastructure will be selected to conduct a trachoma control pilot study (March-April 2001). One county-hospital ophthalmologist and one township ophthalmologist/general practitioner will be in charge of the study. The study will benefit from the participation of the government at both levels and patients will be identified/recruited by trained village PEC workers and township civil affairs officials. It is planned that each ophthalmologist will perform 20 trichiasis operations per day.

V. TIMEFRAME (January 2001 – December 2015)

- First half of 2001: Training of 100 PEC workers
- March-April 2001: Initiation of township trials
- May 2001-Dec.2015: Implementation of antibiotic treatment and surgery

During the implementation phase, the county and townships will set up extra surgical teams in areas where infrastructure is insufficient and where prevalence is high, preferably outside the farming season.

V. AVAILABLE AND NEEDED RESOURCES

Staff: Full use will be made of all available county and township hospital ophthalmologists, as well as village PEC workers.

Technology: Efforts will be made to train technical personnel, standardize surgical procedures, assess surgical capacity and provide guidelines for management of surgical complications.

Equipment: Existing surgical equipment at county and township hospital levels will be used. The acquisition of equipment by village clinics (health centres) will be made possible by collective funding or support from the provincial and country governments and donations from CBM.

Funding: Subject to subsidies expected from the government and the Federation of Disabled Persons, as well as from individual donations, patients fees and international support

VI. IMPLEMENTATION, MONITORING AND SURVEILLANCE

The County Health Bureau, the County Blindness Prevention Office, as well as township governments and township health centres will work in collaboration for the implementation of the project. They will be responsible for coordination between the various parties, mobilization and allocation of available resources (human and material), education and information of patients,

Project monitoring and surveillance will be carried out by the County Health Bureau together with the County Blindness Prevention Office. It will include issues such as the quality of staff training, the effectiveness of surgery, the results of the distribution of antibiotics, cost-effectiveness analysis and sustainable development. A data base and a county-wide trachoma control network will also be established to improve management.

VII. EVALUATION

Regular assessments of the project carried out twice a year are planned to ensure that targets are reached, that treatment is effective and to identify and solve problems when necessary. A final evaluation is planned at the end of the project in 2015.

ANNEX 5

ABOUT THE NEED FOR A STANDARDIZED TRACHOMA GRADING SYSTEM IN CHINA

There are a number of internationally recognized classification methods for trachoma, among which the Mac Callan method is one of the more widely used. It involves the classification of trachoma into four phases: phase I is the early stage of infiltration; phase II is the progressive stage of infiltration; phase III is the scar formation stage; and phase IV is the recovery stage. This is a rather complicated classification and is difficult to master. In 1979, the Ophthalmology Society of the Chinese Medical Association classified trachoma into three phases: phase I involves completely active trachoma; phase II concerns active trachoma plus scar formation; and phase III is the stage of complete scar formation. From a clinical and epidemiological point of view, such classification is more practical. The characteristics of the WHO's new grading system for trachoma are as follows:

1. The grading system is simplified and easily understandable, using the conjunctiva of the upper eyelid as the indicator for determining trachoma infection, which is easier for primary eye care workers to understand and therefore particularly appropriate for epidemiological surveys of trachoma involving large areas in developing countries.

2. In addition to three situations concerning trachoma (TF, TI and TS)•, the grading system includes complications of trachoma, i.e., TT and CO•, thus facilitating the development of therapeutic programmes according to the finding of the survey.

3. WHO recommends that the main indicators of trachoma prevalence in a given areas, as assessed with its grading system, should be as follows:

   i. The prevalence of active trachoma (TF and/or TI) in the under-10 age group, which indicates the prevalence of trachoma infection in the area;

   ii. The prevalence of TI in the under-10 age group, which indicates the severity of trachoma infection in the area. WHO proposed measures, based on the prevalence of TF and TI among children under 10 years of age, to control active trachoma (see table). Children under 10 years of age are taken as an indicator in the development of programmes to control active trachoma since children account for a large proportion of the population in areas with a high prevalence of trachoma, making children suffering from active trachoma the major source of trachoma transmission;

   iii. The prevalence of TS, which indicates the prevalence of trachoma in the past;

   iv. The number of TT cases, which indicates the number of cases requiring prompt surgical intervention to correct entropion;

   v. The prevalence of CO, which indicates the rate of blindness caused by trachoma.

• TF&TI : Trachomatous inflammation - Follicular (TF) & Trachomatous inflammation - Intense (TI)
TS : Trachomatous scarring
TT : Trachomatous trichiasis
CO : Corneal opacity

WHO's measures to control active trachoma

Active trachoma in <10 age-group (%)	Basic treatment	Complementary treatment
TF>20% or TI>5%	Universal use of locally applied antibiotics	Generalized application of antibiotics for severe cases
TF 5%-20%	Universal or selective individual use of locally applied antibiotics	Ditto
TF<5%	Selective individual use of locally applied antibiotics	

❖ ❖ ❖ ❖ ❖
