# Supplementary material for: Assessment of trachoma in suspected endemic areas within 16 provinces in mainland China
Source: PLoS Negl Trop Dis. 2019 Jan 28;13(1):e0007130. doi: 10.1371/journal.pntd.0007130 (PMC6366720; doi:10.1371/journal.pntd.0007130)
Supplement: S2 Document — (PDF) [file pntd.0007130.s004.pdf]

WHO/PBD/GET/00.8
Distr.: General
Original: English

WORLD HEALTH ORGANIZATION
PREVENTION OF BLINDNESS AND DEAFNESS

Guidelines for RAPID ASSESSMENT for blinding trachoma

A.-D. Négrel, H.R. Taylor & S. West

© World Health Organization, 2001

This document is not a formal publication of the World Health Organization (WHO),
and all rights are reserved by the Organization.
The document may, however, be freely reviewed, abstracted, reproduced and translated, in part or in whole, but not for sale nor for use in conjunction with commercial purposes.

The views expressed in documents by named authors
are solely the responsibility of those authors.

Acknowledgements

This document was developed and printed with the support of:

The Edna McConnell Clark Foundation
Pfizer Corporate Philanthropy
The Task Force of the International Agency for the Prevention of Blindness

Technical assistance provided for the development and/or field testing of these guidelines is gratefully acknowledged from the following:

Dr Y. Chami-Khazraji
Ministry of Health, Morocco

Dr H. Fall
Ministry of Health, Social Welfare and Women's Affairs, Banjul, The Gambia

Dr H. Limburg
Bovenkarspel, The Netherlands

Professor D. Mabey
London School of Hygiene and Tropical Medicine, London, England

Dr S. P. Mariotti
Prevention of Blindness and Deafness, World Health Organization, Geneva, Switzerland

Dr R. Pararajasegaram
Prevention of Blindness and Deafness, World Health Organization, Geneva, Switzerland

Dr D. Sacko
Epidemiology Division, Ministry of Health, Bamako, Mali

Dr J.-F. Schémann
African Institute of Tropical Ophthalmology, Bamako, Mali

Trachoma Rapid Assessment

Dr B. Thylefors
Former Director, Prevention of Blindness and Deafness, World Health Organization, Geneva, Switzerland

Table of contents

Guidelines for rapid assessment for blinding trachoma

CHAPTER 1
Introduction

	Page
About trachoma	1
A need for a simple but effective tool to collect information	3
What is Rapid Assessment (RA) ?	4
What Rapid Assessment is not !	6

CHAPTER 2
Preliminary assessment

Overview	9
Designation of a RA coordinator and assignment of reviewers	9
Possible sources of data	10
Review of written reports	10
Interviewing key informants	11
Semi-structured interview	12
Where to collect the information	13
Analysis and validation of available information	15
Interpretation of validated data	17
Outcome on completion of the phase 1	19

CHAPTER 3
Organizing and implementing field visits

Overview	21
Selection of villages to which visits should be made	21
Preparing for the visit	23
Appointing a field coordinator	23
Selecting a team	24
How many teams are necessary ?	24
Training the team	25

Preparing an identification card for trichiasis 26
Preparing the RA forms for data collection 29
Equipment and logistics 30
Timetable 31
Ensuring maximum community cooperation 32
The visit 34
Meeting the community 34
Assessing the pattern of trichiasis in the community 36
Touring the village 38
Steps in the RA for active trachoma 39
Selection of children to be examined 39
Assessing the active trachoma pattern 40
Other activities 42

CHAPTER 4

Analysing data and setting priorities

Overview 43
Data processing 43
Assessing indicators 44
Assessing the trichiasis pattern 44
Assessing the active trachoma pattern 44
Principles in data interpretation 45
Setting priorities 45
Priority ranking of villages for lid surgery 45
Priority ranking of villages for treatment of active trachoma and other interventions 45
What to do with the two priority lists 46
Standardizing assessment teams 47
Rationale 47
Procedures to be followed 47
Comparing the results among the RA teams examinees 48

CHAPTER 5

Rapid assessment for hygiene and environmental factors

Overview 49
Collection of observational data 49
Hygiene: unclean faces in children 52

Environmental factors 53
Community profile for hygiene and environmental factors 54
Unclean faces 54
Environmental factors 55

CONCLUSIONS

ANNEXES

Booklet of data collection forms

Form 1. Village form 60
Form 2. Trichiasis form 62
Form 3. Active trachoma form 64
Form 4. Environmental factors form 67
Form 5. RA summary sheet 69

Foreword

Purposes of the document

This document introduces the concept and methodology of Rapid Assessment (RA) for those seeking to control and eliminate trachoma–related problems through cost-effective interventions.

The purposes of the document are:

- To enable eye-health managers to collect data in order to develop a plan of action based on community needs;
- To make rational use of scarce resources for data collection with a view to optimizing the benefits of trachoma control activities.

The document provides both a checklist of information needed and an approach for obtaining data that will enable planners to identify the existence of trachoma-related problems and plan action to provide solutions.

Using all the data obtained, eye-health managers will be in a position to rank the endemic zones in order of priority and need for control measures. They should also be in a position to predict the number of communities and the size of the population that will require coverage by an active trachoma control or elimination campaign (such as surgery for trichiasis, distribution of topical or systemic antibiotics, or other community-based interventions) for a given level of endemicity.

This document advocates a primary health care approach based on the principles of community participation.

Aims and objectives

- To optimize the use of limited resources through a simplified rapid assessment method for the collection of data.

- To identify and prioritize communities for intervention using the SAFE strategy (Surgery, Antibiotic treatment, Facial cleanliness, Environmental improvement), and in particular to identify communities with active trachoma to implement antibiotic distribution, face cleaning and environmental changes and also those where trichiasis surgery is required.

- To facilitate, after the identification and prioritization of communities, more detailed evaluation of community resources and needs for the implementation of the SAFE strategy.

CHAPTER 1.

INTRODUCTION

1. About trachoma

Trachoma is a common disease in certain developing countries. Close to 150 million persons worldwide suffer from active disease, while an additional 6 million are blind or at risk of visually disabling complications.

Today, trachoma is found in underprivileged communities with poor living conditions where there is little hope of rapid socioeconomic development. The disease is found mainly in remote rural areas of most African countries, in some Eastern Mediterranean countries, and in parts of Central and South America. Trachoma is also endemic in several Asian countries, but there is a lack of up-to-date information from some highly populated countries, including China and India.

The severity of trachoma - and thus the extent of its blinding potential - varies from region to region and community to community. Assessment is required to determine the presence or absence of trachoma as a public health problem and the nature of the interventions to be carried out in a given community in order to eliminate blinding trachoma.

The presence of trachoma is closely related to living standards and hygiene. The prevalence of active disease in communities may change rapidly in the absence of targeted interventions if socioeconomic development occurs.

There is therefore a need to identify, with a fair degree of confidence, the regions and communities that should be given priority in interventions (treatment and prevention).

The evolution of the disease typically occurs in two stages, which are separated by several years or (often) decades:

- **inflammatory (active) trachoma**, diagnosed most often in children; in many settings, girls tend to have more frequent and severe active disease than boys;

- **cicatricial or scarring trachoma**, generally found in adults and leading ultimately to the development of trichiasis; this is often found 3-4 times more commonly in women than in men.

Consequently, when assessing trachoma at the community level, it is important to consider both the active trachoma in children and the potentially blinding complications (i.e. trichiasis) in adults.

In certain circumstances, only mild, non-blinding trachoma is found in a community; if so, cases of active trachoma are seen in children but these are very few or no cases of trichiasis. In such circumstances there is usually no need for mass interventions at the community level and only recognized active cases need to be treated individually.

On the other hand, there are communities where trachoma may have been a severe disease in the past. Very few cases, if any, of active trachoma are identified in children. However, cases of trichiasis in adults are still present in the community and trichiasis surgery needs to be provided without delay.

In longstanding hyperendemic areas, severe active trachoma will be found in children as well as trichiasis and trachoma-related visual impairment in older persons.
Risk factors for a pattern of severe trachoma (i.e: leading to blindness) are well known and easy to identify at the community/family level. They include:

- Lack of facial cleanliness among children, which facilitates the exchange of infected ocular secretions between children and with other family members. Dirty faces also attract flies to the eyes, thus increasing the risk of transmission of trachoma and the development of repeated eye infections.

- Crowding, in circumstances where people live in close physical contact with each other, e.g. sharing the same sleeping space (linen) or bedding.

- Insufficient environmental sanitation, particularly for sewage and garbage disposal at the community level, and the lack of latrines at the family level; these and other factors (such as keeping cattle next to human dwellings) enable flies to breed close to the households.

Consequently, once communities have been prioritized for eyelid surgery or antibiotic distribution, it is important to assess the existing risk factors in order to design appropriate environmental interventions.

2. The need for a simple but effective tool to collect information

As trachoma is closely associated with living standards and hygiene, its epidemiological pattern may change relatively rapidly, even if no specific intervention is carried out but when socioeconomic development occurs in the community. That is why the information obtained to plan/re-plan a trachoma project/intervention should reflect the actual epidemiological pattern existing in the communities concerned. Health planners and managers need a better insight into the current trachoma situation than is normally available from routine information systems.

Managers and planners need to have a fairly quick and cost-effective method for deciding where to put scarce resources for trachoma control.

In order to use those scarce resources in a cost-effective and appropriate manner and to identify and reach the communities most in need of intervention, it is necessary to determine where the highest levels of severe blinding trachoma are found. Thus, for programmatic purposes, a rational, rapid and low-cost method of identifying specific areas/communities likely to have a significant problem of blinding trachoma is needed.

Rapid Assessment (in which health managers review the existing records, interview key informants and make direct "observations" at the community level when necessary) is a way of obtaining this information.

3. What is Rapid Assessment (RA)?

RA is a method of obtaining information about a set of problems in relation to trachoma, in a short period and without a large expenditure of professional time and funds. The term "rapid" refers both to the time spent in the field collecting the data and the time spent analysing these data. This should be the minimum acceptable time to gather current information from which to develop a plan of action expeditiously.

The basic principle is to collect at low cost the maximum of pertinent information in the minimum of time.

RA represents the first step in the process of identifying the communities that should be prioritized for planning and implementing trachoma interventions. It should be considered as an operational tool, developed to help decision-makers to identify and rank for priority the communities most affected by trachoma for treatment/action. RA is a practical way of determining rapidly whether or not blinding trachoma is endemic in a given region or community.

RA data will facilitate the planning of trachoma control activities through the identification of high-risk zones where large-scale interventions are indicated.

Information for RA may be obtained:

- **from existing written documents;**
- **through interviews or group discussions;**
- **through direct observations made during field visits and eye examination performed in selected age groups.**

RA is based on community participation.

In order to obtain the blocks of information needed, RA requires the systematic completion of the following steps:

- Organize a first phase of investigation. This rather static or passive phase (mainly desk work) represents a preliminary assessment and consists of:

  a) looking up, gathering and reviewing all existing documentation concerning trachoma and its complications, as well as socio-economic information, from the area to be studied;

  b) validating and analysing the information gathered on completion of this step;

  c) deciding what additional information is needed to complement the data obtained so far;

- Organize a second phase of investigation - the dynamic or active phase (mainly field work) - and conduct visits to selected communities in order to confirm or complete the necessary information.

These steps will be described in the following chapters.

FIGURE 1

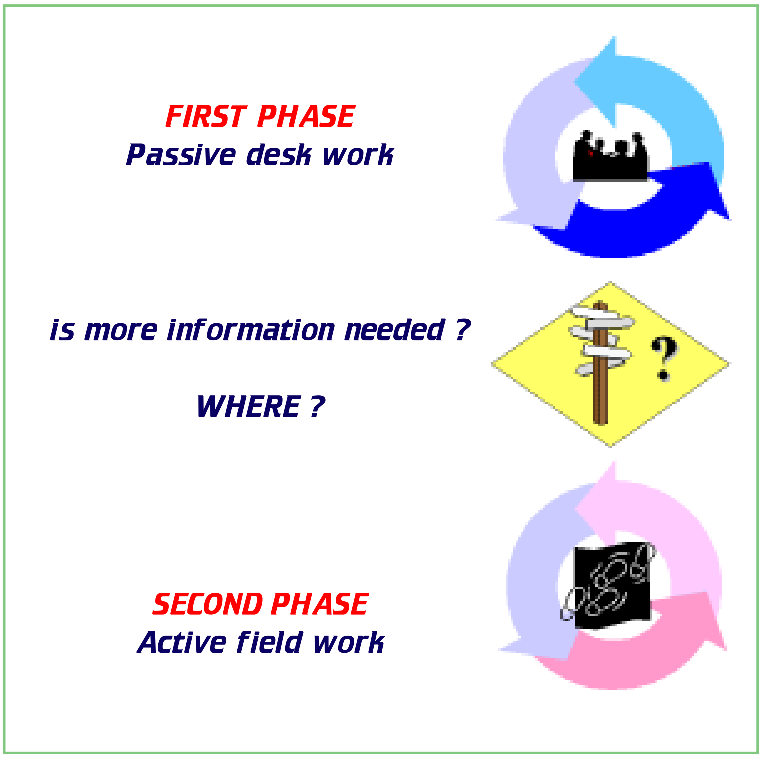

4. What Rapid Assessment is not !

RA cannot quantify the size of the trachoma problem in the community. However, after a problem has been identified and given priorities by planners, a detailed survey may be necessary to provide baseline data.

RA is not a sound basis for comparison of the burden of trachoma in different areas, except for use in ranking regions or communities.

Limitations of Rapid Assessment

- RA can be used as a specific tool to indicate what the trachoma-related problems are in a given community, but not the exact number of persons affected by trachoma.

- RA does not provide an accurate population-based epidemiological assessment. It should be used to prioritize/ rank communities or areas for large-scale interventions.
- RA does not and cannot replace epidemiological surveys to assess the magnitude of the trachoma problem. It is not suitable for monitoring or surveillance, and does not provide a baseline for evaluation of interventions; more accurate methods are needed for these tasks.

Rapid Assessment can therefore be used to:

- Identify areas/communities where trachoma is endemic;
- Determine whether or not trachoma is a blinding disease
- Prioritize communities with blinding trachoma for immediate intervention.
- RA is, and should remain, the beginning of a process for the collection of information in order to prepare a plan of action against trachoma.

General principles

- **Do not collect too much or irrelevant information or data that are difficult to analyse;**
- **Tailor or adapt investigations to reflect local conditions and specific situations;**
- **Involve the community.**

CHAPTER 2.

PRELIMINARY ASSESSMENT

1. Overview

As the final goal is the **elimination of blinding trachoma**, the information of immediate interest concerns essentially the identification, review, analysis and validation of information relating to the existence of a problem of **trichiasis and blindness due to trachoma in selected areas/communities/regions.**

Staff will need to be assigned to the project, typically a coordinator, supported by reviewers/interviewers to help in identifying and reviewing critically existing local information.

2. Designation of a RA coordinator and assignment of reviewers

The preliminary activities will usually be carried out by a **group** of several (two or three) **reviewers/interviewers** under the responsibility and supervision of a **national or provincial RA coordinator.**

Attitudes and skills required for RA coordinator

- **Commitment to and perseverance in discovering and examining written records;**
- **Willingness to learn from local people;**
- **Willingness to use local resources;**
- **Readiness to listen carefully during both informal conversations and group discussions;**
- **Awareness and sensitivity to everything that can be directly observed;**
- **Use of common sense in analysing the information.**

3. Possible sources of data

The necessary information on locations where trachoma may be a problem can usually be found from:

- various types of **reports**, e.g. those from regions where trichiasis is prevalent (hospital data on trichiasis surgery);
- other anecdotal sources, such as **interviews** with key informants and **focus group discussions**.

3.1. Review of written reports

The term "**review**" in these guidelines refers to a systematic process for collecting existing data on **trachoma, eye diseases** and **eye surgery**, and analysing the various types of information gathered through this process.

It is recognized that though written reports may have limitations, they remain a major source of information. The reviewer(s) must keep in mind that the purpose of RA is to get **relevant** information **quickly**. Many of the documents/reports relating to eye diseases or eye surgery may be bulky, but actually contain very little - if any - of relevant information in relation to trachoma. To attempt to read the whole of each document would therefore be counterproductive. The available documents should be **scanned** purposefully; the relevant portions should be noted and then read in full detail.

Types of records, reports and other "**documented evidence**" that might prove useful are listed in the box below.

Useful information sources

- **Surveys on blindness and/or trachoma, if undertaken fairly recently;**
- **Other disease surveillance or health status investigations where blindness and trichiasis may have been reported;**

Outpatient clinic and hospital records on trachoma, trichiasis and trichiasis surgery. The health facility concerned should have a reasonably well defined catchment area and population. As trachoma occurs in rural, often remote areas where access to health care is difficult, the absence of cases in these centres does not necessarily mean that trachoma is not a problem.

3.2. Interviewing key informants

Interviews with selected key informants are often very appropriate during this first phase of RA for gathering information useful to a better appreciation of the situation.

Key informants can be chosen in the two following groups:

- **Health care or related professional informants** likely to have some practical knowledge of trachoma, eye diseases and blindness;
- **Lay informants**, i.e. people who, because of their official leadership positions,have direct or indirect access to information about community rather than individual problems.

FIGURE 2

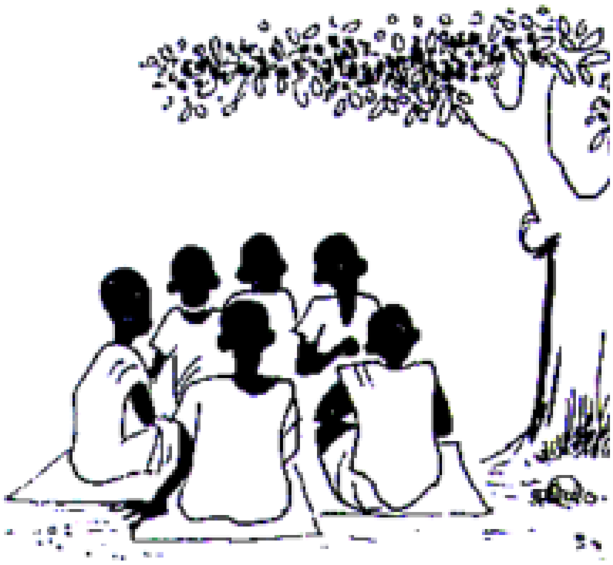

Key informants : likely persons

At **national** and **provincial** levels, the use of key informants and/or a focus group approach can provide information on regions with severe trachoma and trichiasis. Key informants include:

- provincial directors of health;
- ophthalmic personnel;
- social and health service personnel;
- representatives of United Nations agencies;
- representatives of nongovernmental developmental organizations (GDOs);
- other people with knowledge based on experience.

At **district** and **community** levels, it will be useful to make direct contact with:

- community leaders (elected officials, religious leaders, women's group leaders,heads of community organizations, traditional healers , teachers, etc.);
- local health workers (mobile team);
- local health authorities;
- traditional healers;
- the staff of nongovernmental organizations operating in the area.

3.3. Semi-structured interview

Semi-structured interview techniques may provide considerable anecdotal evidence of a "hidden" problem and, will often provide the basis for visiting communities at risk. A semi-structured interview is a **guided discussion** which should be conducted in a rather informal way.

- A **checklist** of trachoma-related questions should be prepared in advance.

- Most questions need to be **open-ended.** This encourages informants to answer in a free-ranging style.

- Sufficient time should be allowed for establishing a **relaxed atmosphere** and asking all the relevant questions.

- Informants should be given the opportunity to introduce **aspects not anticipated** by the interviewer.

3.4. Where to collect the information?

The approach to be followed depends mainly on the levels at which data are expected to be required, e.g. **national, provincial or district levels.**

When the decision-makers need to have information at the national level, the following situations should be considered:

- The country is known to have been **endemic in the past** and it has **no operational programme for the prevention of blindness**. In such circumstances, there is usually no up-to-date or directly accessible information concerning trachoma-related blindness. The question is whether blinding trachoma is still present in some communities or has disappeared, at least as a public health problem. It is useful, in such a situation, to adopt and develop a **"top-down" approach:**

- The search for information should start first at the **national level**, with the **consent and participation of the Ministry of Health staff concerned**. At this stage, the objective is to identify provinces/regions where blinding trachoma is "**likely to be endemic**".

- Once the regions have been identified, the same procedures should be applied to identify **candidate districts** where trachoma is "**likely to be endemic**".

- Within identified districts, the same procedures should then be applied to identify **villages/communities that are "likely to be endemic".**

A programme for the prevention of blindness has been operating in the country for quite a long time, and the regions or districts with trachoma have been fairly well identified by the health authorities:

- RA procedures should be simplified by gathering information directly at the level of the districts long-known to be endemic for blinding trachoma.

- These districts will then be considered as an **opportunisic "entry point"** for further investigations, in order to identify villages/communities needing to be rapidly assessed.

FIGURE 3

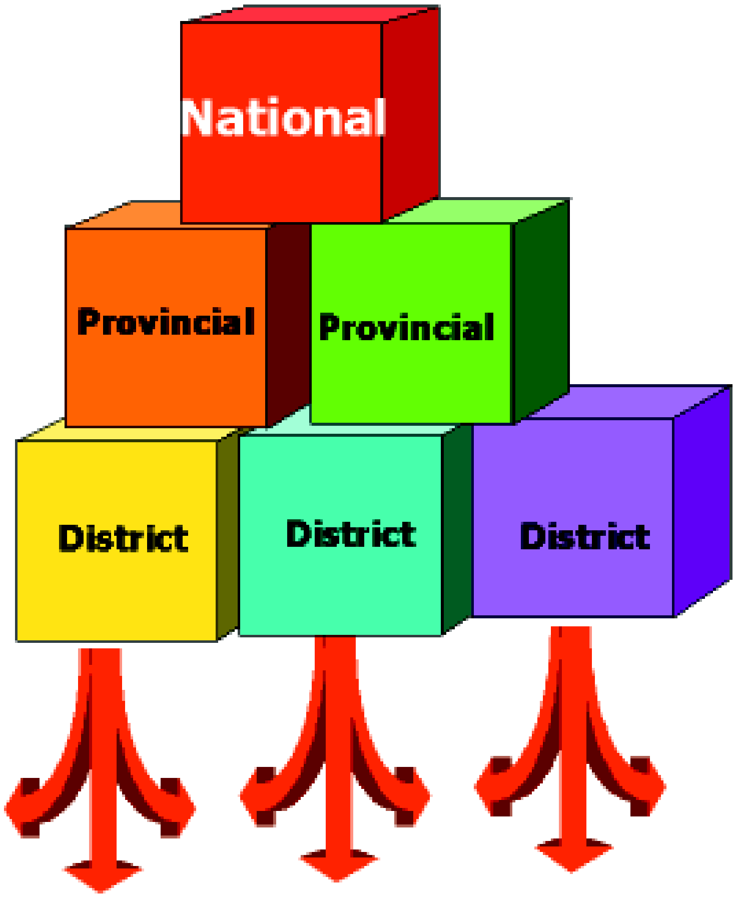

VILLAGES/COMMUNITIES

3.5. Analysis and validation of available information

It is the *quality*, not the quantity, of information that is important.

***Discussions* between the coordinator and the reviewers about what information is relevant, up-to-date, and reliable are critical.**

Simple methods should be used to analyse and validate the obtained data.

To make sense of the data, it is necessary to **group** the different findings:

- Data from various documents and responses from informants should be grouped in **blocks**.For example, trachoma information collected from written documents or informants concerning trichiasis can be kept on white cards, information related to inflammatory trachoma on yellow cards, and so on.

- Using this approach, data collection and analysis from the first phase of investigation can in fact proceed simultaneously.

In RA, it is important **not** to rely on a single source of information, and it is preferable to have **confirmatory evidence from different sources** (two or more documents, information from several knowledgeable persons).

- If several sources of information are available, data from one source should be **cross checked** with data from at least one other source. An important step is to compare the information from key informants with information obtained from the review of documents.

- If there are large discrepancies in the sets of data, the points of difference should be noted and a decision taken about how to explain these conflicting findings, including proceeding to Phase 2 of the Rapid Assessment.

FIGURE 4

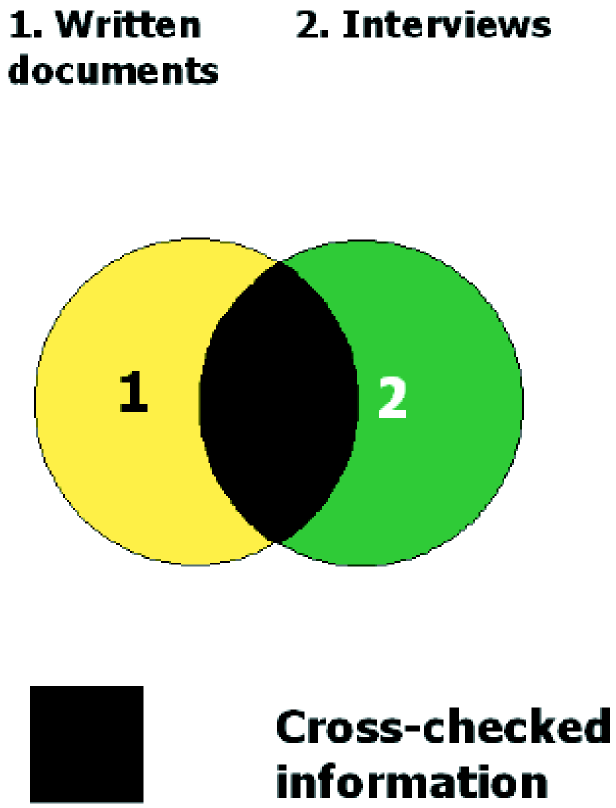

- Once relevant information has been identified, it should immediately be recorded in as much detail as possible:

- The **title** of the document and other identification details, such as **author(s) and date,** should be noted and an **abstract** should be prepared;

- Documents providing information **on more than 7-10 years old should be reviewed with caution**, especially if the districts concerned have experienced substantial socioeconomic development of significant changes in living standards or lifestyles in recent years. Such "old" data may, however, still be useful to indicate the possible presence of residual disease. This is especially true for data on trichiasis. Hospital registries or other records of trichiasis surgery can be very useful in this regard.

All accepted ("validated") information should be used in order to:

- Confirm the "**presence"/"probable presence**" or the "**absence/"probable absence**" of blinding trachoma in the zones concerned;

- Identify precisely **provinces/regions/districts** likely to have blinding trachoma. All "suspected" and confirmed areas should be methodically and exhaustively **pinpointed on a map**;

- List **adjacent/neighbouring areas** carefully and systematically, so that further investigations/discussions can be undertaken (especially when the RA procedures described above have not yielded sound information for these areas).

4. *Interpretation of validated data*

At this stage the RA reviewers may face three different situations:

Trachoma-related data exist (the data may be **positive** or **negative)**

- If the analysis provides **definite evidence** of blinding trachoma in a well identified zone, the province/region/district/community should be considered as **endemic**. However, **further confirmation of the severity and a quantitative assessment may be needed for planning purposes**. In such a situation, some villages may be selected at a later stage and included in Phase 2 of investigations (see Chapter 3).

- If the analysis demonstrates **the non-existence** of trachoma in a zone, no more assessment is needed for this particular area. Areas and populations where there is no evidence and/or those that are "unlikely" to have blinding trachoma (based on the availability of good socioeconomic indicators), should as a rule, **be omitted from further assessment**. Although there may be isolated cases of trachoma in such settings, it is very unlikely that there is a significant public health problem needing urgent intervention.

Some incomplete, outdated trachoma-related data have been reviewed and more or less validated

- This information may provide **insufficient knowledge/ evidence** to place the regions or districts concerned in a definite category.

- Some villages may then need to be selected and included in **Phase 2 of investigations** (field visits), in order to arrive at a more definite assessment.

No direct trachoma-related information is available

- The team of investigators should then look carefully for other available information, e.g. **indicators on living and hygienic standards** in the provinces/communities concerned. Selected key informants (not necessary the same as those already interviewed) can be very useful for this exercise.

- An **analysis of similarity** should then be carried out, taking fully into account the data from neighbouring districts where a more accurate assessment of the trachoma situation is available.

  - Provinces or districts should be grouped according to socioeconomic, geographic and ethnic characteristics so that **adjacent areas** without any trachoma-related information can be compared with those identified by the preliminary approach.

  - Where the socioeconomic and ethnic characteristics of adjacent districts are considered very similar by the team of investigators and the key informants, then the areas should be considered as **"alike" areas**, by extrapolation from the available information as to the likely presence or absence of blinding trachoma between zones/ communities identified as similar.

* Depending on a review of this new set of data, the province, region or district will be considered as either:

  - "**Likely to be endemic**" for blinding trachoma, if the **area is known for poor living and hygienic standards** and if **blinding trachoma is prevalent in an adjacent district**. Further confirmation will be needed and these areas will be included in the second phase of investigations.

  - "**Unlikely to be endemic**" for blinding trachoma if **living and hygienic standards are apparently reasonably** good and if there is no particular reason to suspect the presence of trachomatous blindness. No more RA is needed for this particular area.

* Before concluding that a province, region or district should be targeted for intervention based on analyses of similarity, further validation by field work is always necessary.

5. Outcome on completion of Phase 1

At this stage the country can be mapped into three types of areas:

- Areas or regions **free from blinding trachoma**;
- Areas **with blinding trachoma** where the epidemiological pattern is well known (a fairly rare situation);
- Areas "likely to be endemic", but of **uncertain status.**

Before the areas that are "likely to be endemic" can be ranked by priority for trachoma control, **additional data must be generated**.

The steps described in Chapter 3 should be followed to select villages/ communities to which visits should be made in order to:

- make direct observations;
- identify cases of trichiasis;
- examine a sample of resident children.

These additional data will enhance the capability of the RA to rank communities.

FIGURE 5

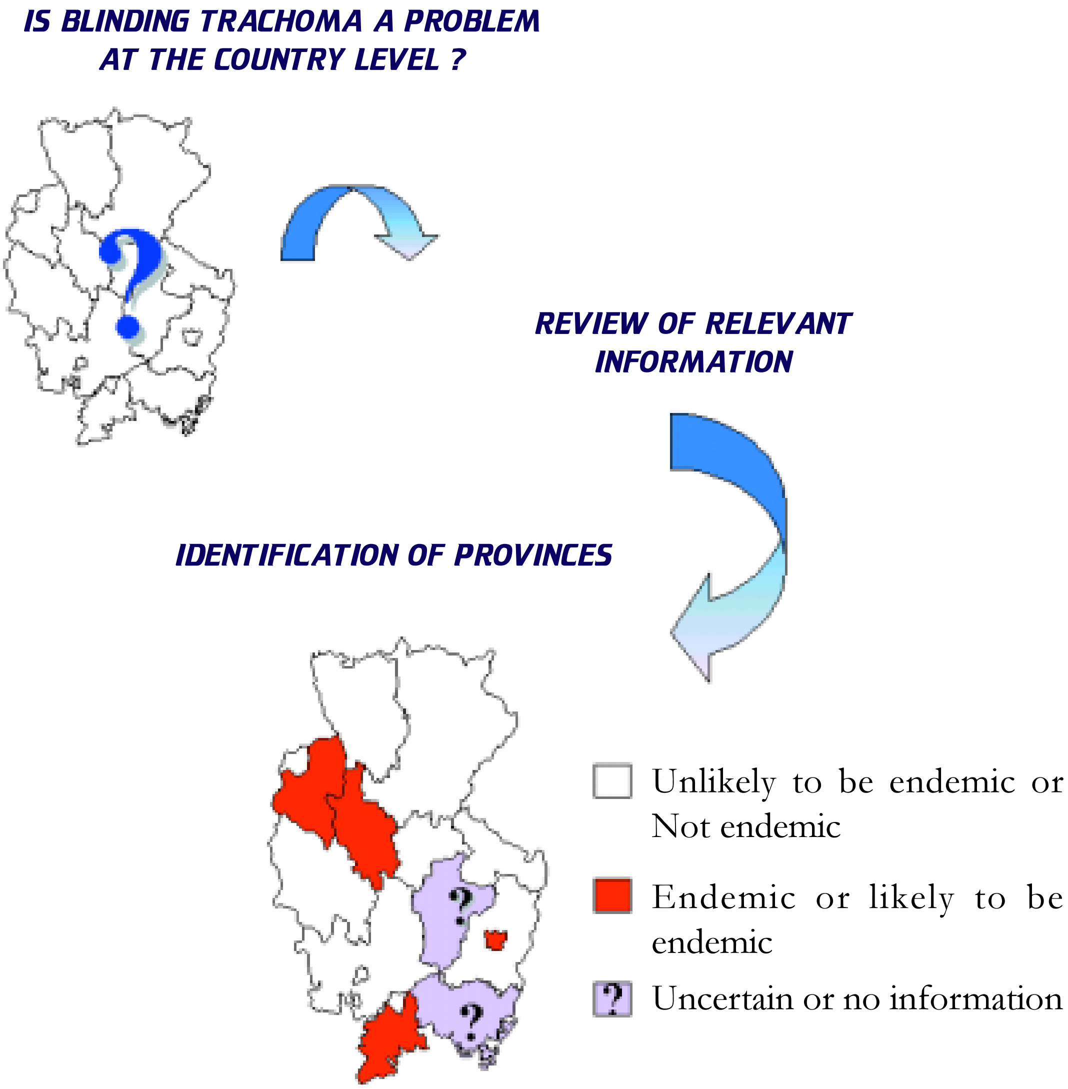

CHAPTER 3.

ORGANIZING AND IMPLEMENTING FIELD VISITS

1. Overview

The aim of the field visits is to ascertain, by a new set of RA procedures applied to **those most at risk,** whether or not trachoma is present in a specific area and, if so, to obtain a reasonable assessment of its severity and a general idea of the level of endemicity.

The proper organization of the field visits requires careful consideration and planning. Therefore, before starting the field work, much **preparatory work** needs to be done. All the administrative and organizational steps described in this Chapter must be planned and carried out.

The RA visits can take time !

Make sure you are not in a rush !

2. Selection of villages to which visits should be made

The objective is to "select" villages/communities where direct investigations should be undertaken to demonstrate clearly/definitely whether blinding trachoma is present or not and if present, its severity.

For these purposes **a convenience sample** should be selected **deliberately choosing communities where trachoma is likely to be present**. In practice, as RA will be carried out preferentially in districts and communities where blinding trachoma is more likely to be found, villages should be selected from among the most **socioeconomically disadvantaged**.

Preference for RA will be given to villages/community in which at least one of the criteria listed in the following box applies.

Choosing communities/villages

- **Uncertain situation or suspicion of trachoma, based on a previous review or the analysis of similarity;**
- **Evidence of trachoma from previous reports or from key informants (using the field visit to validate the information derived from the previous review);**
- **Isolated communities of less than 500 people, with special attention to minorities and marginalized mobile or migrant population groups or tribes);**
- **No easy or permanent access to water;**
- **Primary health care services are weak, irregular or non-existent;**
- **No school in the community.**

The RA approach uses an optimally biased selection of a strictly limited number of villages/communities deliberately targeting those at higher risk.

The proportion of the total number of communities in the area to be appraised is not a main concern in these **deliberately biased selection procedures**.

Initially, when several districts are involved and RA is being conducted on a wide scale throughout the provinces, at least **three and usually no more than seven villages** may be investigated in each district. Later on, in order to fill in the gaps and gradually obtain the needed information for ongoing trachoma control activities, all the villages in the area concerned can be assessed in turn.

A list of villages to be visited should be compiled and village names should be pinpointed on a map.

Trachoma Rapid Assessment

As it is common in many areas where trachoma is prevalent to find several villages or hamlets with the same name in the same district, it is advisable, whenever feasible, to keep a precise record of the **latitude** and **longitude** of all selected villages right from the start. This information can be obtained from locally available documents and should be included in the corresponding section of the Village Record Form (see Annex, Form 1). This preliminary task will be particularly useful for programme managers using a **geographical information system (GIS).**

3. Preparing for the visit

3.1. Appointing a field coordinator

A **"field coordinator"** should be designated. This person could be the national RA coordinator or some other, e.g. a local district health officer or someone on his or her staff, with the necessary organizational background.

Among other tasks, the field coordinator will

- decide **when** the field assessment will start, and the **time required** to survey each selected area;
- decide the order in which the districts and selected communities will be assessed;
- **select**, **train** and organize the field team(s);
- be responsible for the **logistics.**

Attitudes and skills required for RA field coordinator

- **Capacity to be a leader;**
- **Capacity to be a trainer;**
- **Willingness to learn from local people and use local resources;**
- **Careful listener during both informal conversations and group discussions (communication skills);**
- **Cross-cultural sensitivity;**

- **Readiness to conform to procedures/guidelines to be followed;**
- **Awareness and sensitivity to everything that can be directly observed;**
- **Use of common sense in analysing the information.**

3.2.Selecting a team

The coordinator should select and train the field team members. Basically, a RA survey team will optimally comprise **four members:**

- The **team leader**, who should have the necessary skills to assess not only the clinical situation, but also additional problems, when visiting the villages. Professional experience is particularly useful to the team leader, e.g. as a general practitioner or an ophthalmic assistant familiar with the WHO simplified trachoma grading system.1

- An **assistant** who should be **a trained ophthalmic nurse** able to perform eye examinations and use the WHO simplified trachoma grading system.

- A **health or social worker/interpreter** with communication skills, who needs to have a thorough understanding of local customs and culture. This person should not only help to overcome problems caused by ignorance of local customs and insensitivity to them; but also to prevent problems from arising in the future. He or she should have a thorough knowledge of the local language and customs, and ideally should already be known and appreciated by the community leaders. (The local district public health staff or administrator can help in the recruitment of this staff member.)

- A **driver.**

3.3.How many teams are necessary?

For each country/province, decision needs to be taken on the number of RA teams to be trained and used. While it may seem attractive to

use a large number of teams composed of personnel selected at the district or community level, this is relatively inefficient because of the imperative **need for standardized training and supervision** (see Chapter 4). Moreover, more validation will be required, as performance of each team will have to be validated.

To avoid these problems, it is recommended that the number of field teams be limited to a small number (2-4) who can be trained at the provincial level. These teams can benefit from better training and supervision. During the field work, they can be strengthened by health personnel from the district level and by village residents when necessary.

3.4.Training the team

The staff concerned should be well prepared, through a training programme, so that the rapid assessment field procedures can be conducted efficiently and in the shortest possible time and in the same manner in different places.

Principles in Rapid Assessment training

- **The training should be essentially task-oriented;**
- **Regular classroom (few) and field sessions (many) should be held;**
- **All problems likely to be encountered should be discussed/addressed. Free discussions and comments should be encouraged;**
- **Special emphasis should be placed on improving practical communication skills;**
- **At the end of the training period, a practical test could be given to ensure that those who complete the training have attained the required level of skills.**

The coordinator should pay special attention to ensuring that the eye examiners are using the **WHO simplified trachoma grading system** correctly and in a similar fashion, and that it has been fully understood (see Chapter 4).

As the evaluation of reliability is crucial, an attempt should be made to assess the consistency of examiners' finding in order to reduce variations to a minimum before field work begins. The methodology for setting up and conducting a simple reliability study is well described in the WHO/EMCF document entitled **"Primary health care level management of trachoma"** (WHO/PBL/93.33, available on request from the WHO office for the Prevention of Blindness and Deafness). By such techniques, the quality of the field investigators can be raised considerably, so that **a fair degree of reliability** of results can be achieved.

Ideally, the practical training should be continued in the first few villages included in the RA. The team members can then be left to work on their own, once the field coordinator is sure of their competence.

As long as the team is in contact with the community, it should be **supervised.** Relevant and continuing training to reinforce both the quality of work and the motivation of the staff should also form part of the training programme.

3.5.Preparing an identification card for trichiasis

An illustrated identification card should be prepared **to be shown to village residents** to help them understand what trichiasis is. Together with appropriate oral descriptions, the card will help the RA team to contact the persons with trichiasis using the information provided by residents, without having to conduct a comprehensive screening of the village population.

Different types of illustrations and pictures can be useful in specific settings. They can make explanation clearer. Unfortunately, they can also confuse. Often people who are not used to looking at pictures just do not understand what they represent.

FIGURE 6

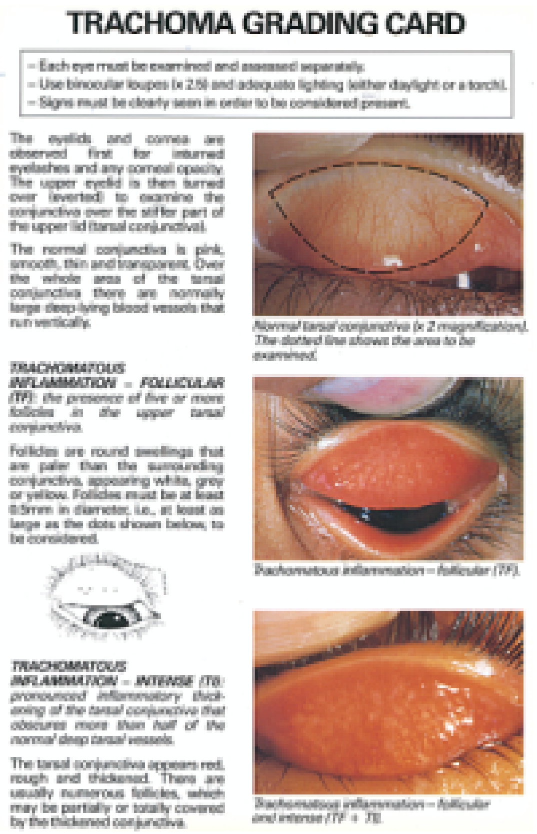
TRACHOMA GRADING CARD

- Each eye must be examined and assessed separately.
- Use binocular loupes (x 2.5) and adequate lighting (either daylight or a torch).
- Signs must be clearly seen in order to be considered present.

The eyelids and cornea are observed first for inturned eyelashes and any corneal opacity. The upper eyelid is then turned over (everted) to examine the conjunctiva over the stiffer part of the upper lid (tarsal conjunctiva).

The normal conjunctiva is pink, smooth, thin and transparent. Over the whole area of the tarsal conjunctiva there are normally large deep-lying blood vessels that run vertically.

Normal tarsal conjunctiva (x 2 magnification). The dotted line shows the area to be examined.

***TRACHOMATOUS INFLAMMATION – FOLLICULAR (TF):** the presence of five or more follicles in the upper tarsal conjunctiva.*

Follicles are round swellings that are paler than the surrounding conjunctiva, appearing white, grey or yellow. Follicles must be at least 0.5mm in diameter, i.e., at least as large as the dots shown below, to be considered.

Trachomatous inflammation – follicular (TF).

***TRACHOMATOUS INFLAMMATION – INTENSE (TI):** pronounced inflammatory thickening of the tarsal conjunctiva that obscures more than half of the normal deep tarsal vessels.*

The tarsal conjunctiva appears red, rough and thickened. There are usually numerous follicles, which may be partially or totally covered by the thickened conjunctiva.

Trachomatous inflammation – follicular and intense (TF + TI).

FIGURE 7

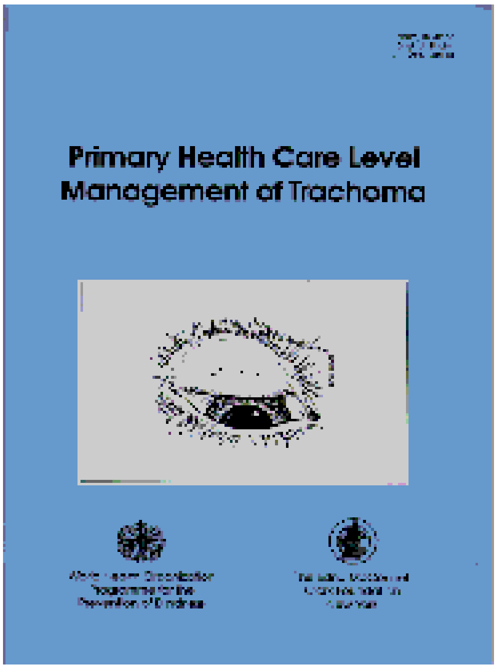

Candidate illustration(s) should be **field-tested** for appropriateness and ease of understanding by the rural population before they are adopted for use.

FIGURE 8

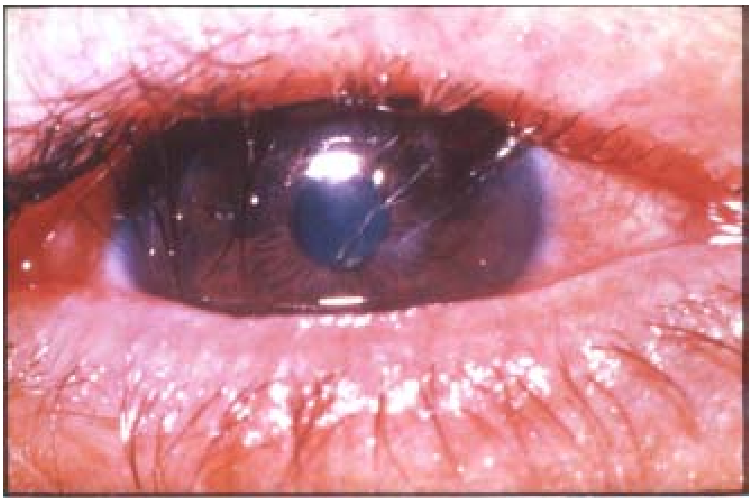

Which illustration ?

- **Photographs:** While these can be very useful, they can also be confusing. Moreover, many practical problems can arise in the preparation of the identification card, e.g. difficulties in obtaining suitable photographs and in reproducing them cheaply.

- **Drawings** (shaded or line drawings): Drawings may be the most useful way of illustrating trichiasis. The drawing can be prepared to show only the features that are necessary and "educationally" important: the face of an elderly woman, both eyes and the direction of the eyelashes, and/or the use of tweezers). Yet they must be realistic enough for the village residents to recognize what is shown.

- **Symbolic or stylized drawings:** This type of drawing is the easiest to reproduce, but it requires a great skill in drawing. Also, it may not be easily understood by the people.

- **Cross-section**: A cross-section of the eyeball and the lid is certainly a powerful way of showing the direction of the

eyelashes towards the cornea. But understanding a cross-section is not an innate skill. People who are not used to looking at cross-sections (or pictures) will have much difficulty in understanding them.

- **Paintings**: More difficult to prepare, "artistic" paintings may distract the attention of people from the message to be transmitted. Too much art or detail can be distracting.

> **!! Remember !!**
>
> ***Pictures are not understood automatically by everybody***
>
> ***Keep the illustration simple***
>
> ***Test your illustration***

3.6.Preparing the RA forms for data collection

A series of five record forms should be completed for each village/community visited. Five forms are proposed in the Annex to these Guidelines:

- **A form for the identification of the village and its characteristics (Form 1);**
- **A form for the assessment of trichiasis (Form 2)**
- **A form for the assessment of "active trachoma" and "dirty faces" (Form 3)**
- **A form for the assessment of environmental factors (optional procedure) (Form 4);**
- **A form entitled RA summary sheet (Form 5).**

A complete and detailed record should be kept of all the observations made, such as:

- **the results of the lid/eye examinations performed, the names of the persons examined and the case numbers and names (if available) of all "suspected cases" of trichiasis;**

- **the number and percentage of children with active trachoma.**

The RA model forms presented in the annex can be adapted by the field coordinator to match local situations and/or administrative constraints**.** The field coordinator should arrange for **field-testing** of all the forms.

The coordinator should provide the team leader with a sufficient number of forms for completion of the field work.

3.7.Equipment and logistics

Very little is required by way of **equipment and supplies**.
The trachoma examiner and the team will need some simple equipment:

- **loupes** (x 2.5 magnification);
- **torches** and **batteries;**
- an adequate quantity of **record forms;**
- **drugs** such as tetracycline 1% eye ointment or, if applicable, azithromycin.

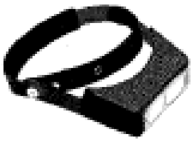
Loupes (X2.5)

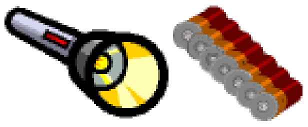
Torches&Batteries

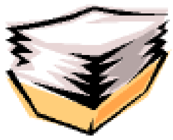
Forms

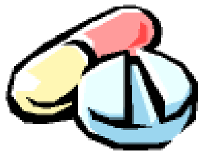
Drugs

During field visits, the team leader or other team members may have **to treat routine medical problems.** They should therefore take aspirin and antimalarial tablets with them to maybe use for treatment;

supplies may be left behind with a local health worker as a public relations gesture.

Maps and **stationery** may also need to be purchased.

Means of transport for people and equipment are essential in order to reach marginalized people, as is often the case with communities with trachoma. Transport will often be a big problem and need to be carefully considered. A vehicle (preferably with **four-wheel drive**) will be required to allow the team to move easily from village to village. It should be **allocated to the team for the full period of the fieldwork**. Running costs for the vehicle, i.e., **maintenance** and **gasoline,** should be budgeted for.

Accommodation and cooking facilities also need to be arranged in advance. The field team will be in the field for several days or even weeks. Members will therefore have to be paid an allowance for food and lodging.

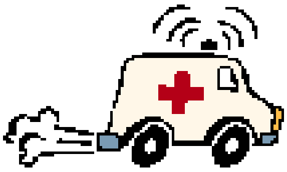
Transportation

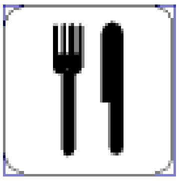
Cooking

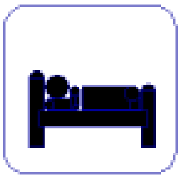
Accomodations

3.8.Timetable

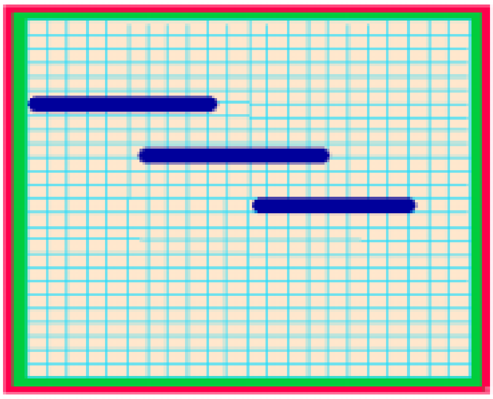

A timetable should be drawn up to indicate precisely the **order** of the field visits and the dates on which they will be undertaken. The visits should keep to specified dates and be completed within the **scheduled time.**

Before an investigation is undertaken, the **local conditions** need to be carefully checked and clearly understood to ensure that there is no special local event that could hamper the team visit, e.g**. a local festival or market day.** For example, the movements of the population in an area where regular markets are held should be taken into consideration. Fieldwork during the rainy season may run into transportation and communication problems.

The **climate and season** are important factors (the activity and mobility of the population differ according to the season).

The **accessibility of villages** is an important consideration in fixing the timetable for investigation. Extra time should be allowed for possible mishaps (flooded rivers, impassable tracks, etc.).

It is wise to plan to visit **one village per working day.**

- The team should aim to arrive at the village in **the late afternoon** of one day in order to inform and mobilize the community. The RA should be carried out on the following morning and the team should then move on to the next village in the afternoon.

- In some cases, **the team may need to arrive on the day** of the field work. In such circumstances, the team should aim to arrive very early, in order to find most of the residents in the village, before they start their daily activities.

3.9.Ensuring maximum community cooperation

RA is principally a **community-oriented activity.** Without the participation of the community, the objectives of RA will never be achieved**.**

To **ensure maximum cooperation**, the local authorities (administration, village leaders) should be **notified in advance** of the impending visit;

The notification, made through the appropriate (locally available) channels of communication, should clarify the points listed in the box below.

FIGURE 9

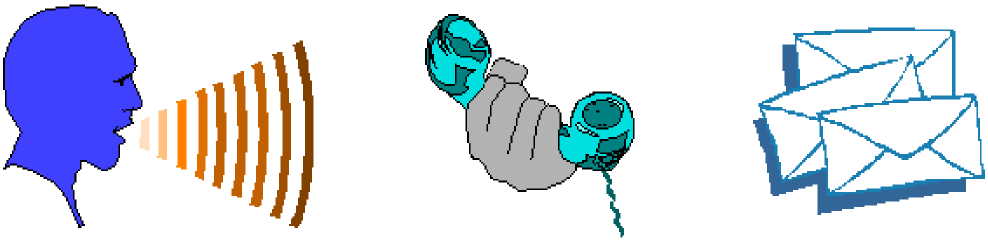

Notification in advance

Informing the Community

- **Clarify the purpose of the RA (e.g. identification of specific eye-related problems in the community).**
- **Mention the precise date and time of arrival of the team.**
- **List the possible ways in which community life could be disrupted by the visit.**
- **Specify the practical arrangements needed to make the visit a success (e.g. wide participation of the community, a place to hold discussions with community leaders and members of the community).**
- **Obtain any clearance required from the local authorities in advance.**

4. The visit

4.1.Meeting the community

As soon as the village is reached, the village leaders, together with any other residents who could be of help, must **be clearly briefed on the purpose of the visit** (even if they have already been notified during the preparation phase) and encouraged to assist the team.

FIGURE 10

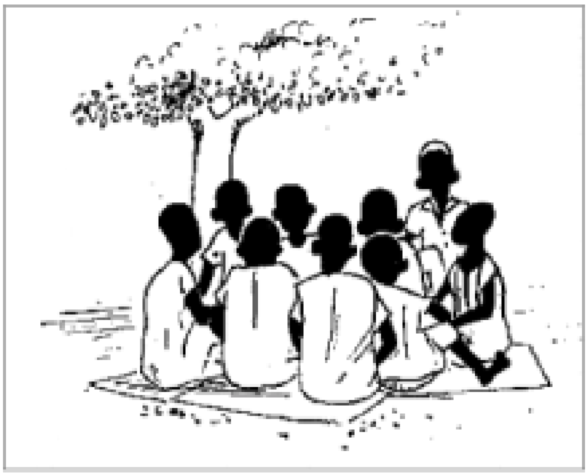

Initial contact with the community leaders

- **The team leader must explain in detail what is going to be done, and for what reason.**
- **All the field procedures to be applied should be described, discussed at length and finally accepted by the community.**
- **All questions should be answered.**
- **People should also be informed that there are plans to control trachoma in the community if the disease is recognized to be of public health importance, and that all eligible residents will be treated free of charge.**

It is particularly important to explain that not all households or children will be examined in a systematic manner. Nevertheless, people who will not be examined but who have an eye problem will be offered an eye examination to determine if there is a need for treatment and referral.

- This preliminary briefing of the community leaders should be extended to a larger mixed audience (both women and men), including traditional healers, if there are any in the community concerned.

- During this larger group discussion, **general information** should be actively sought by the team, especially in relation to the village infrastructure and access to health care facilities, including trichiasis surgery. **The information collected should be noted on the Village Form** (Form 1).

FIGURE 11

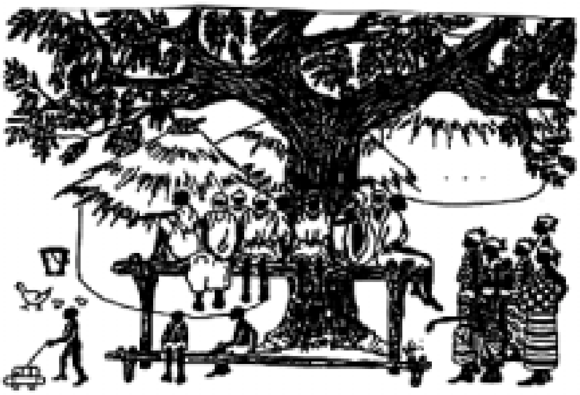

Collecting information of interest

How many people live in the community?

An estimate of the population should be made. Only an approximate figure will be needed when analysing the data, and it is not necessary to do a detailed census for the RA. A previous census or villages sources can be used.

- **Is there a primary health care dispensary or a primary health care worker in the community? If not, how long does it take to gain access to this type of service?**

- **What is the availability of trichiasis surgery? Where? When?**

4.2.Assessing the pattern of trichiasis in the community

This step is essential to assess the epidemiological pattern of trichiasis in the community. It should take advantage of the presence of the wide range of participants in this group discussion.

The practical objectives are to identify and to contact as many as possible, if not all, of the persons with trichiasis living in the community.

Trichiasis will be assessed through a series of simple questions, followed by the identification by the community members of persons "likely to have" trichiasis.

Using the specially prepared and tested "picture" in order to facilitate the recognition of the lid disease by at least some of the participants, a group discussion will be held to ask the questions presented in the picture below.

FIGURE 12

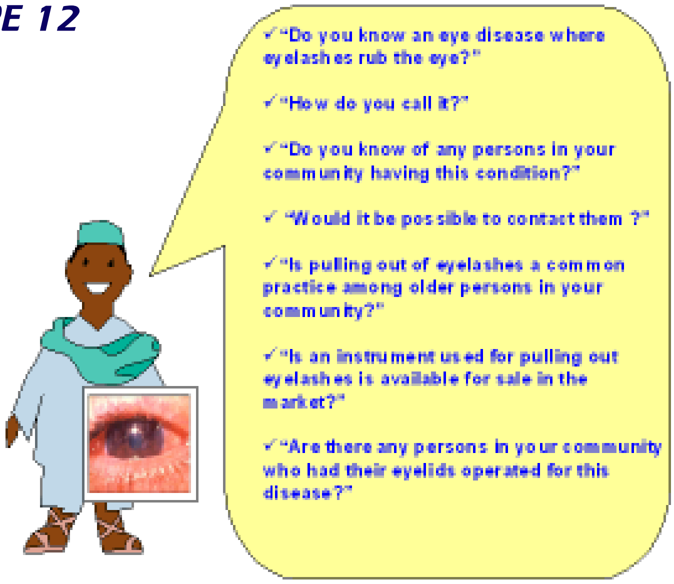

Examples of questions about trichiasis

- Do you know an eye disease where the eyelashes rub the eye?
- What do you call it?
- Do you know anyone in your community who has this condition?
- Would it be possible to contact them?
- Is pulling out the eyelashes a common practice among older persons in your community?
- Is an instrument used for pulling out eyelashes available for sale in the market
- Has anyone in your community had their eyelids operated on for this disease?

After this group discussion, all persons identified by the community or having **"likely trichiasis"** should be **contacted** before the team leaves.

An eye examination should be carried out in every case of "suspected" trichiasis.

House visits should be arranged, if need be, to examine people who do not come to the team voluntarily or who have mobility problems.

The team should first confirm that suspected cases are in fact cases of trichiasis. The confirmation should be carried out in accordance with the **WHO simplified trachoma grading system.**

Remember

Even one eyelash rubbing the eye is trichiasis !

The team should then ascertain local **capacity to perform trichiasis surgery** (if possible at the village level).

Finally, **a list of patients** to be operated on should be prepared, and practical arrangements for referral should be made if lid surgery cannot be performed in the village.

The information will be **recorded** on the Trichiasis Form (see Annex, Form 2).

Suspected cases who could not be contacted during the team's visit should be listed separately so that they can be contacted and examined at the earliest opportunity.

The number of people with trichiasis indicates the level of priority need for the provision of surgical services for lid correction

4.3.Touring the village

At the end of the group discussion the team, accompanied by representatives of the community, should go round the village.

Such a tour allows the team to make observations to help identify sections of the village from which children should be selected for examination. It should be remembered that RA is optimally biased towards the most disadvantaged parts of the village.

Since the main concerns are the environmental conditions that make the presence of trachoma possible, many problems such as those listed below can be easily observed during the tour.

FIGURE 13

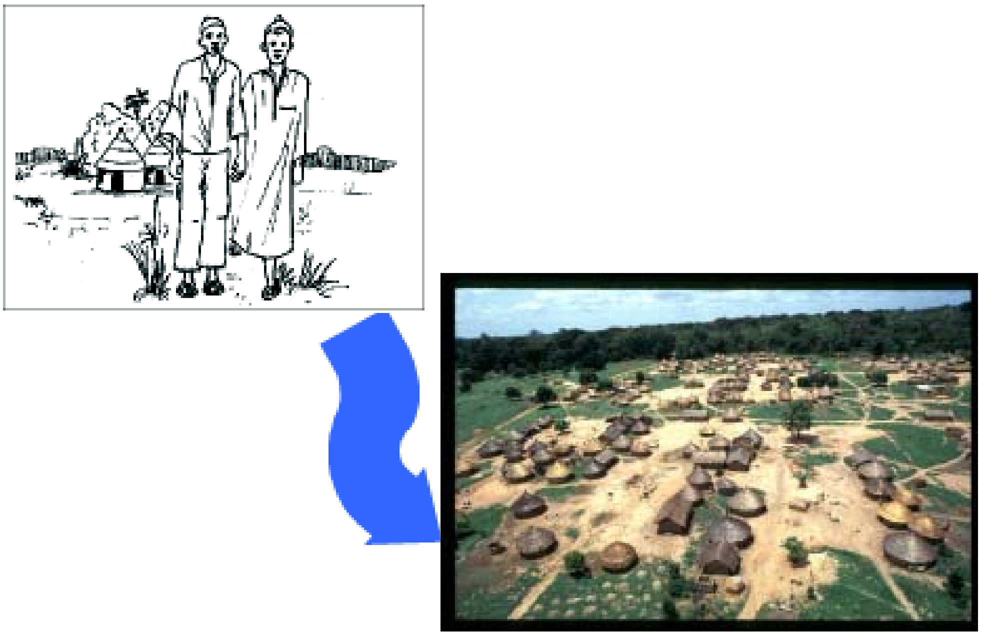

Observations regarding the "environment"

- **Housing conditions**
- **availability of latrines and their use**
- **animal pens close to the houses**
- **garbage pit close to the houses**
- **high fly populations**
- **Solid waste disposal**
- **Availability of water (wells, pumps, ponds, rivers)**
- **Availability of road communications/access**
- **Other factors**

These observations (optional) should be recorded in the **"Notes and Remarks"** section of the Village Form (see Annex, Form 1).

The clinical assessment of children should be carried out in what appear to be the **most socioeconomically deprived sections/ neighbourhoods** of the village, based on lack of water, lack of basic sanitation, and/or evidence of crowded living conditions.

5. Steps in the RA for active trachoma

5.1.Selection of children to be examined

Fifty (50) children (1-9 years old) should be targeted from the selected households to provide evidence of the presence or absence of active infection in the community. At least half of the 50 children should be preschool children.

Children to be included in the rapid clinical assessment should be selected from at least **15-20 households/compounds**.

In areas where villages consist of continuous households with well structured neighbourhoods, it will be sufficient to survey 15-20 households in one section or in each neighbourhood.

In areas where villages consist of widely spaced households, **scattered** households should be assessed until the required sample size of 50 children is reached.

If a selected village does not have enough children to reach the sample size requirements, **examine all the children present** and make a note to that effect in the "Notes and Remarks" section of the Active Trachoma Form (see Annex, Form 3).

5.2. Assessing the active trachoma pattern

The objective of the RA is to ascertain by a standardized examination of the **everted upper eyelid** whether active trachoma is present and, if so, to obtain **a "worse case estimate"** of its endemic level in the most disadvantaged households.

The examinations can be conducted either:

- in a **central meeting point** (accepted by the majority of the participants) where the children have been gathered together; or

- **at home**. It is usually easier to find children in their homes. Such an approach takes longer but is always more effective to reduce the number of absentees and enhance coverage. When the population is very scattered, the only option is to make home visits.

Each eye should be examined **separately**. The result of the **worse eye** should be recorded in the Village Record Form (see Annex, Form 1).

The examination should be made according to the **WHO simplified trachoma grading system,** using binocular loupes with **x 2.5 magnification**. If the examination takes place indoors, a good torch is needed. If outdoor daylight is sufficient, the patient should face the sun.

The examination can be performed either standing or with both the patient and the examiner seated on facing each other. Instructions are given in the Trachoma Grading Card and all the examination

procedures are described in full in the WHO document **"Primary health care level management of trachoma" (WHO/PBL/93.33**).

Before and after the examination of each child, the examiner should systematically clean her/his hands to prevent transmission, and to set an example.

FIGURE 14

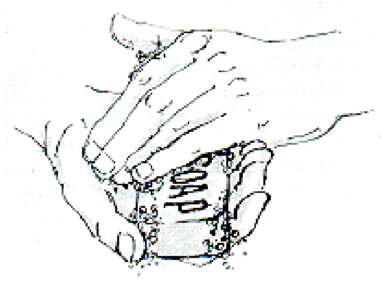

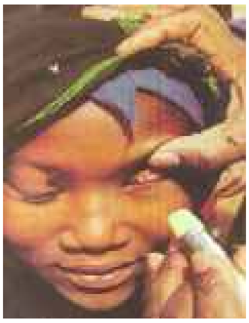

Cleaning of the hands before and after the eye examination

Only **follicular trachomatous inflammation (TF**) and/**or intense trachomatous inflammation (TI)** should be noted during this examination. A child is recorded as **"positive" if TF and/or TI** is present (put a tick under "yes" in the appropriate section of the Active Trachoma Form (Form 3). Otherwise, the child is recorded as **"negative"**, even if there are signs of trachomatous scarring (put a tick under "no" in the appropriate section of the form).

Doing the surveys during school hours in the local school will provide biased information, as only the older children who are less likely to have trachoma are present, and the most disadvantaged children often do not attend.

FIGURE 15

Remember

TF :
the presence of FIVE or more follicles, at least 0.5 mm in the upper tarsal conjunctiva

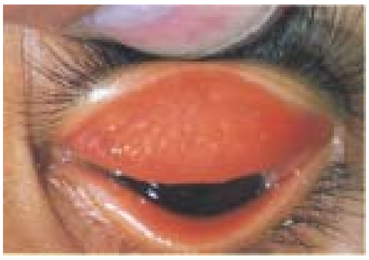

Remember

TI :
pronounced inflammatory thickening of the tarsal conjunctiva that obscures more than half of the normal tarsal vessels

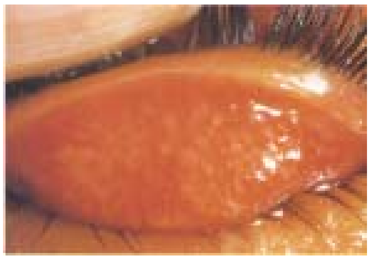

5.3.Other activities

It is important to ensure that the team offers **additional services** that provide some obvious benefit to the people participating in the RA exercise.

While there are time constraints during the RA, there will often be enough time to **examine all persons who present for an eye examination**. In areas where medical care is absent or scarce, the provision of simple, low-cost but effective care and treatment services for other conditions (pain, fever, etc.), including referral when necessary, should be planned, budgeted and implemented.

The RA team should be prepared to treat or refer such persons.

Such extra activities will help provide good relations, and represent a way of attracting and sustaining community participation.

Remember

There can be no survey without service !

1 *Thylefors B et al. A simple system for the assessment of trachoma and its complications.* Bulletin of the World Health Organization, *1997, 64(4): 477-483.*

CHAPTER 4.

ANALYSING DATA AND SETTING PRIORITIES

1. Overview

Collected data only become really useful when they have been processed and analysed. The RA results should then enable the coordinator to make rational use of scarce resources with a view to optimizing the benefits of trachoma control activities. The Coordinator must ensure that the different teams are using uniform methodology to collect the RA data.
In this chapter, only data used to identify and prioritize communities for surgical and treatment interventions are considered.

2. Data processing

Data processing can be done **at the end of each village assessment:**

- Tally the total number of persons in the village (estimated from a recent census or village leaders). There is no need for the RA team to do comprehensive enumeration of the residents;
- Tally the number of persons with “confirmed” trichiasis and the number of persons with “suspected” trichiasis;
- Tally the total number of children examined;
- Tally the number of children with TF and/or TI.

The advantage of this approach is that recording mistakes can be **corrected** immediately.

“Hand tallies” can summarize the data in each category rapidly and produce a concise statement of the main findings.

A **hand calculator** can be used to convert the reported figures into percentages.

A computer can be used for more sophisticated analysis if desired.

3. Assessment indicators

Three indicators should be considered in arriving at a final ranking of the villages and setting priorities in an equitable manner:

- The number of cases of trichiasis
- The "rough prevalence estimate" of trichiasis
- The percentage of active trachoma among children.

These data will be available from the data collection forms (see Annex).

3.1..Assessing the trichiasis pattern

Two measures are of interest:

- **The absolute number of cases of trichiasis** which represents the **surgical load** for the village;

- **The prevalence of trichiasis in the community** which provides data on the proportion of the population affected by trichiasis.

This indicator is computed as follows:

Number of trichiasis cases* examined in the community divided by the estimated population of the community

3.2..Assessing the active trachoma pattern

The percentage is calculated as follows:

Number of children with TF and/or TI divided by the number of children examined (at least 50)

** If the team collected reliable data on "suspected cases" during the field visit, then use the number of cases examined plus the number of "suspected cases" divided by the estimated population*

4. Principles in data interpretation

It is important to remember that the communities selected **do not represent a random sample** and that for the assessment of the degree of magnitude of "active" trachoma, the children examined **may not be** representative of the village children. Interpreting and extrapolating RA results as if they "represent" the population as a whole **is misleading**.

Once this **limitation** is understood, an attempt should be made :

- to delineate the high-risk zones for blinding trachoma, and priority areas for full implementation of the SAFE strategy.
- to collect/summarize additional data on personal hygiene and the environment.

5. Setting priorities

For each community the data sets should be analysed independently for trichiasis and active trachoma.

5.1.Priority ranking of villages for lid surgery

The highest priority should be given to communities with **the largest number** of trichiasis and suspected trichiasis cases. These may be large villages, they may **not** have the highest prevalences, but will have the **greatest concentration of cases**.

5.2.Priority ranking of villages for treatment of active trachoma and other interventions

For implementation of the "full" SAFE strategy, villages should be prioritized according to the level of active trachoma. List the villages in the area in order of highest percentage of active trachoma among children examined.

The villages with the highest percentages will be ranked first on the priority list.

Two separate lists of villages should be prepared in order of priority:

- **For trichiasis: Highest priority should be given to communities with the largest number of cases of unoperated trichiasis and "suspected" cases**

- **For active trachoma: The villages with the highest percentages should be ranked first on the priority list.**

5.3.What to do with the two priority lists ?

Trichiasis

Based on the priority ranking of the villages, the coordinator will need to organize the effective delivery of surgical services. This will include the following actions:

- Ascertain the availability of surgeons and equipment;
- Check the completeness of the list of patients to be operated on;
- Contact and examine people identified as "likely trichiasis" cases as soon as possible;
- Make the necessary local arrangements for a surgical team to visit the village, where possible;
- Otherwise, arrange transport of all patients to the nearest facility where trichiasis surgery is available;
- Ensure coordination between referral and the availability of services.

All the steps in surgical procedures are described in the WHO/Edna McConnell Clark Foundation manual entitled "Trichiasis surgery for trachoma: The bilamellar tarsal rotation procedure" (document WHO/PBL/93.29)

Active trachoma

Again based on the priority ranking of the villages, the coordinator will need to organize the distribution of an available antibiotic, using the appropriate health infrastructure in the area and according to the locally established treatment strategy.

6. Standardizing the assessment teams

6.1 .Rationale

Programme managers need to be sure that the information from the RA on which they base their decisions is accurate.

It is therefore important to know, before undertaking the RA, that all team members -and particularly the trachoma examiners- are using the procedures in a similar fashion.

Evaluation of reliability is an essential step that must be taken before any RA. The reliability study ensures that the examiners always grade the signs of trachoma correctly. The examiners need to be reassessed periodically.

6.2.Procedures to be followed

It is important to follow standardized procedures.

All the examiners must understand and use the WHO simplified grading system in a similar fashion. The easiest way to check the reliability of all examiners working in different RA field teams is to arrange for each examiner to grade trachoma on the same individuals. At the end of a series of examinations, these grades can be tallied and the extent to which the results agree with, say, the trainers' grading can be compared.

The process is described in the WHO document entitled **"Primary health care level management of trachoma"** (WHO/PBL/93.33).

6.3.Comparing the results among the RA teams examiners

The two series of data (i.e. on trichiasis and active trachoma) reported by the different examiners should be subjected to an **"agreement/ disagreement"** analysis, to ensure that the estimates reported by each examiner **eventually result in equivalent decisions on priority or need for intervention.**

If the agreement between the examiner and the trainer's grading for each category of data is **around 80% or higher**, the RA team can be relied upon.

However, if the agreement percentage is lower, it will be necessary to see where the problem lies by analysing the type of disagreement (please refer to the above-mentioned document WHO/PBL/93.33).

In these particular circumstances the concerned staff members must be retrained.

* If the team collected reliable data on "suspected cases" during the field visit, then use the number of cases examined plus the number of "suspected cases" divided by the estimated population

# CHAPTER 5.

RAPID ASSESSMENT FOR HYGIENE AND ENVIRONMENTAL FACTORS

1. Overview

After identification of communities with a trachoma problem and prioritization for control activities, a more comprehensive assessment of the status of the communities in relation to risk factors and availability of resources will be required in order to implement the SAFE strategy. The implementation of the F (facial cleanliness) and E (environmental improvement) components in priority villages requires information about current hygiene and the village environment around which programmes can be built. Trachoma is more common in children with "unclean" faces, so an assessment of the problems of unclean faces should be made. Trachoma is less common in houses with latrines, so an assessment of the presence of latrines (or the use of latrines) should be done.

2. Collection of observational data

Simple observations by the RA team can be the basis to collect information on hygiene and environmental factors.

There are three possible ways in which the observational data can be collected. The choice of method is best left to the RA coordinator. Each approach has its advantages and disadvantages as described below:

Approach 1

In the first approach, the RA for hygiene and environmental factors is carried out at the same time as RA for trachoma. Observations can be made at the same households where the children graded for trachoma live.

Advantages:

- The team is already in the village for RA, so the additional effort is minimal.
- Data on trachoma in disadvantaged children match observational data on disadvantaged households.

Disadvantages:

- Data are collected in every village, even those with low priority;
- Data collection requires house-to-house visits for observation (a central site approach cannot be used to collect data at the household level);
- The data collected cannot be used as baseline observations for full evaluation, because the sample of disadvantaged neighbourhoods observed is deliberately biased.

Approach 2

In this second approach, the RA for hygiene and environmental factors is carried out in a second stage. Observations should be made in households in the most disadvantaged parts of the village, as described for trachoma RA. These can be the households of families already surveyed, or different households.

Advantages:

- Data are collected only in the villages already prioritized for the control programme (fewer data need to be collected);
- Teams revisit the prioritized villages to continue building relations with village residents.

Disadvantages:

- Data collection requires a second visit (but this can become

an advantage in villages where trachoma programmes will be implemented (see above!);

- The data collected cannot be used as baseline observations for full evaluation because the sample of disadvantaged neighbourhoods observed is deliberately biased.

Approach 3

In the third approach, the observations are collected as part of the baseline survey in the context of future programme evaluation. Households must be selected as a valid statistical sample of the whole village. (The calculation of epidemiologically valid samples is not described in this document).

Advantages:

- The data collected can be used for evaluation purposes;
- Data are collected only in selected villages (although they may not be collected in every village in the programme area, unless all of them are part of the baseline survey)

Disadvantages:

- Data collection depends on the timing of the baseline survey, so information needed to design the control programme could be delayed.

Whichever approach is chosen by the RA team, observations on children and environmental conditions around the houses should be standardized.

A **record** (see Annex, Form 4) should be kept of **all observations** made, such as:

- Selected information concerning the main characteristics of the village/community;

- Number and percentage of children examined with an unclean face;
- Number and percentage of households more than half-an-hour's walk from a functioning water source;
- Number and percentage of households without a "functioning latrine";
- Number and percentage of households exposed to garbage disposal sites, animal pens and other fly breeding sites.

3. Hygiene: unclean faces in children

FIGURE 19
Unclean face

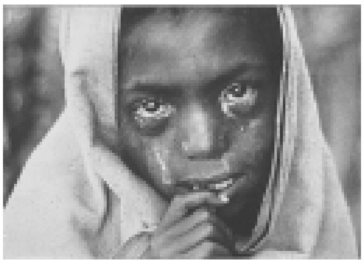

A "clean face" is:

The presence of "sleep" (or ocular discharge) around the eyes

The presence of nasal discharge on the upper lip or cheeks

If any sign of sleep or discharge is present the face is considered "unclean"

Children **must be examined in their household** because they are often cleaned up for a visit to a central site. Therefore, observing children's faces at a central site may underestimate the proportion of unclean faces. Alternatively, if children start crying at the central examination site, they may present with nasal discharge and weeping eyes from crying. Then observations are biased to overestimate unclean faces.

The cleanliness of children's faces should be observed at home

Observers of "unclean" and "clean" faces should be standardized before RA begins.

At least **50 children** (aged 1-9 years old) should be observed for unclean faces drawn from at least 20 households.

4. Environmental factors

Environmental factors thought to be related to trachoma and amenable to intervention will depend on the local situation. They may include some of the following:

FIGURE 20

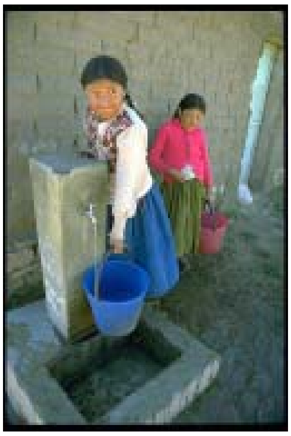

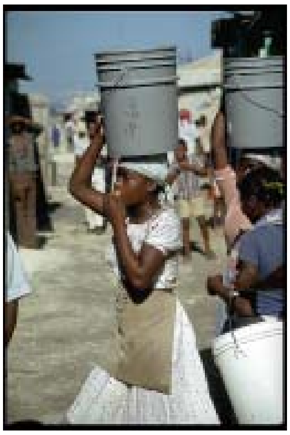

- **Distance from the house to a water source** (in metres, or in time needed to walk from home to the water source);

FIGURE 21

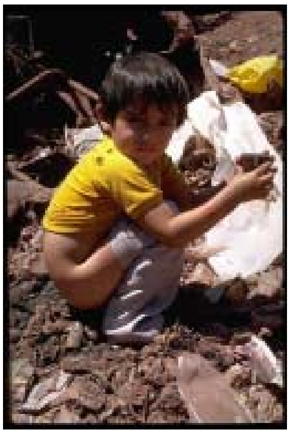

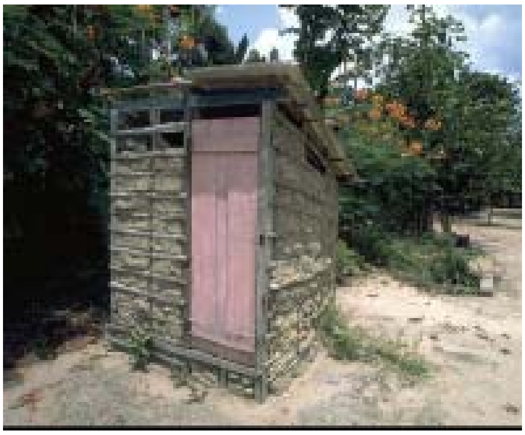

- **Presence of latrines** (and evidence of recent use)/**Absence of latrines**

FIGURE 22

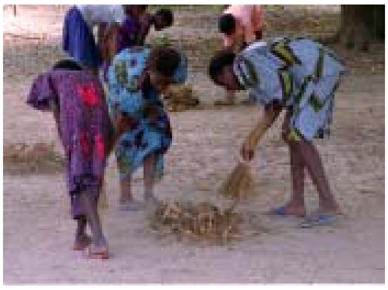

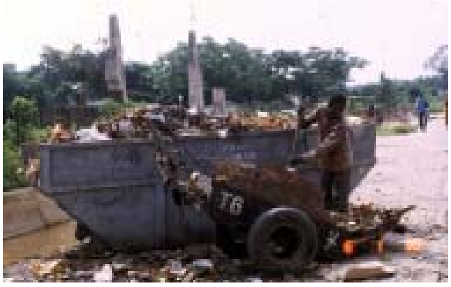

- Evidence of **solid waste or garbage** (excluding animal pens) close to the house (within 20 metres);

- Presence of **animal pens** within 20 metres of the house.

At least 20 households (preferably more: 50 households would be best) should be observed for environmental factors.

The data can be filled in on a form (Form 4) for each household surveyed.

Each local RA team can add additional observations or questions felt to be important for trachoma in its area, as long as definitions are standardized.

5. Community profile for hygiene and environmental factors

5.1. "Unclean faces"

The percentage of children with "unclean faces" can be calculated as:

**The number of children with an unclean face divided by the total number of children observed**

This may be either:

- the proportion in disadvantaged areas of the village; or

- a prevalence estimate for the village, depending on the approach chosen.

The proportion of unclean faces in children gives information on how important face-cleaning is in the community, and if current practices simply need to be extended

5.2. Environmental factors

The environmental factors can be summarized to provide a **profile** either of the most disadvantaged areas of the village, or of the community as a whole, depending on the approach chosen.

Depending on the factors observed, the summary might include the following:

- **The proportion of households more than half-an-hour's walk (or over 4 km) from a functioning water source.** Such data may indicate an urgent need to provide functional water sources for the community, with health education on use of water for hygiene purposes.

- **The proportion of households without a (functioning) latrine.** Such data may suggest a programme to build latrines in the village, or a programme to increase appropriate use of existing latrines (e.g. use by children).

- **The proportion of households with solid waste, garbage or an animal pen close to the house.** Such information suggest that a programme is needed for hygienic garbage, and human waste disposal, to decrease fly breeding sites. If a large proportion of houses have animal pens close to the house, they may be at especially high risk for trachoma/trichiasis, and programmes to protect them may need to be developed.

CONCLUSIONS

Using rapid assessment data

- RA data will indicate the distribution of trachoma in each province/district. Additionally, they will determine the need for further rapid assessment (to "fill the gaps") or full epidemiological assessment to provide a broader picture of the trachoma situation, baseline data or projected numbers of persons with the disease.

- The coordinator will be able to provide more accurate figures on the number of communities (including their population sizes) requiring active public health intervention(s).

- The coordinator will be in a position to rank districts/ communities in order of priority of need for control measures according to the different components of the SAFE strategy. Nation-wide or province-wide planning of control measures will become possible.

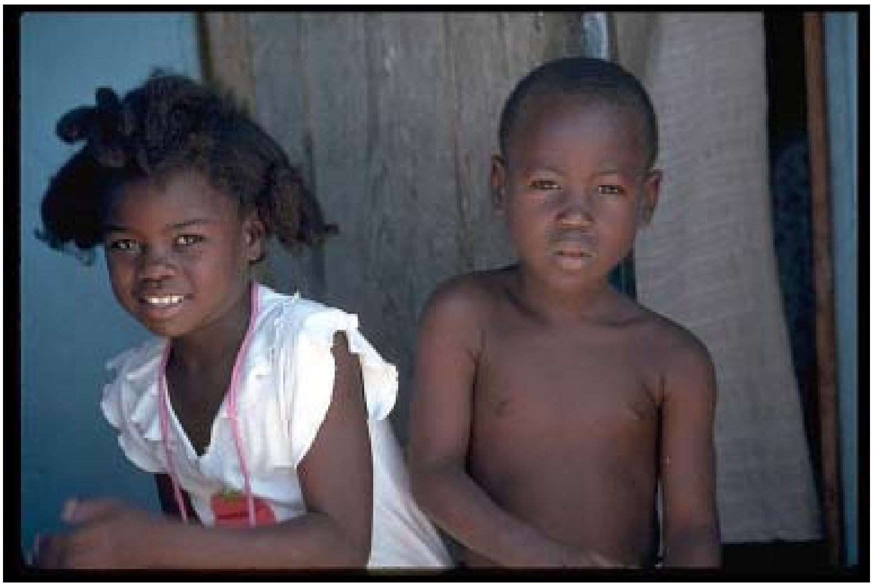

TRACHOMA GRADING CARD

- Each eye must be examined and assessed separately.
- Use binocular loupes (x 2.5) and adequate lighting (either daylight or a torch).
- Signs must be clearly seen in order to be considered present.

The eyelids and cornea are observed first for inturned eyelashes and any corneal opacity. The upper eyelid is then turned over (everted) to examine the conjunctiva over the stiffer part of the upper lid (tarsal conjunctiva).

The normal conjunctiva is pink, smooth, thin and transparent. Over the whole area of the tarsal conjunctiva there are normally large deep-lying blood vessels that run vertically.

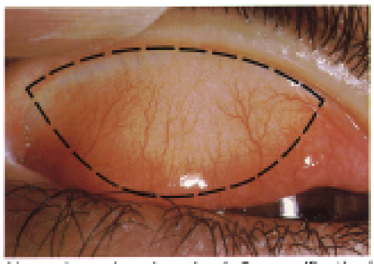

Normal tarsal conjunctiva (x 2 magnification). The dotted line shows the area to be examined.

TRACHOMATOUS INFLAMMATION – FOLLICULAR (TF): *the presence of five or more follicles in the upper tarsal conjunctiva.*

Follicles are round swellings that are paler than the surrounding conjunctiva, appearing white, grey or yellow. Follicles must be at least 0.5mm in diameter, i.e., at least as large as the dots shown below, to be considered.

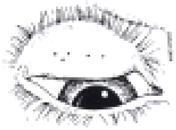

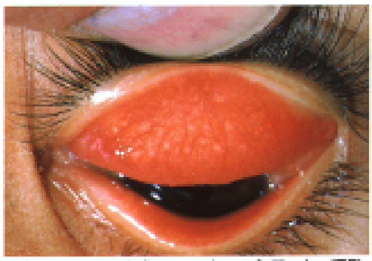

Trachomatous inflammation – follicular (TF).

TRACHOMATOUS INFLAMMATION – INTENSE (TI): *pronounced inflammatory thickening of the tarsal conjunctiva that obscures more than half of the normal deep tarsal vessels.*

The tarsal conjunctiva appears red, rough and thickened. There are usually numerous follicles, which may be partially or totally covered by the thickened conjunctiva.

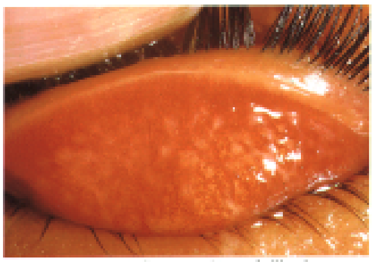

Trachomatous inflammation – follicular and intense (TF + TI).

***TRACHOMATOUS SCARRING (TS)**: the presence of scarring in the tarsal conjunctiva.*

Scars are easily visible as white lines, bands, or sheets in the tarsal conjunctiva. They are glistening and fibrous in appearance. Scarring, especially diffuse fibrosis, may obscure the tarsal blood vessels.

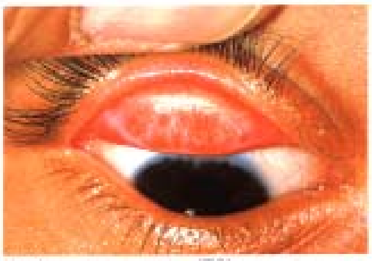

Trachomatous scarring (TS)

***TRACHOMATOUS TRICHIASIS (TT)**: at least one eyelash rubs on the eyeball.*

Evidence of recent removal of inturned eyelashes should also be graded as trichiasis.

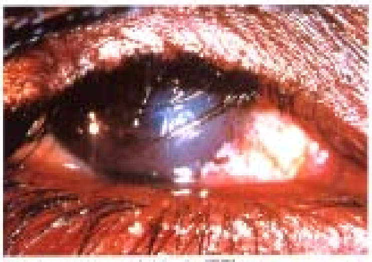

Trachomatous trichiasis (TT)

***CORNEAL OPACITY (CO)**: easily visible corneal opacity over the pupil.*

The pupil margin is blurred viewed through the opacity. Such corneal opacities cause significant visual impairment (less than 6/18 or 0.3 vision), and therefore visual acuity should be measured if possible.

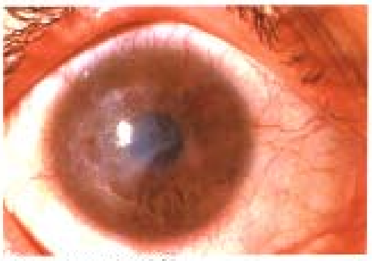

Corneal opacity (CO)

TF:– give topical treatment (e.g. tetracycline 1%).
TI:– give topical and consider systemic treatment.
TT:– refer for eyelid surgery.

WORLD HEALTH ORGANIZATION
PREVENTION OF BLINDNESS AND DEAFNESS

Support from the partners of the WHO Alliance for the Global Elimination of Trachoma is acknowledged.

ANNEXES

RAPID ASSESSMENT FOR TRACHOMA

BOOKLET
of
DATA COLLECTION FORMS

This booklet of forms has been prepared as a set of templates to be photocopied for fieldwork use.

The full set consists of five different forms:

- **FORM 1: Village identification form**
- **FORM 2: Trichiasis form**
- **FORM 3: Active trachoma form**
- **FORM 4: Environmental factors form**
- **FORM 5: Summary sheet**

Each form contains a "Notes and remarks" section to allow field workers to collect any useful additional information.

Form 1

VILLAGE FORM

IDENTIFICATION

**Date**: ...../...../..... (Day/Month/Year)

**Province**:	**District**:
**Village**:	
**Latitude**:[1]	**Longitude**:

**Estimated population of the village**:[2]

**Name(s) of community leader(s)**: • • •

**Team leader**: •

1 Complete this section if a geographical information system (GIS) is available and in use and if a global positioning system (GPS) is available.
2 The figure will be used for assessment of the prevalence of trichiasis.

Form 1 (Continued)

VILLAGE FACILITIES

Village infrastructure	Accessibility [1] (travel time)		
	Less than 30 minutes	30 minutes or more but less than 2 hours	2 hours or more
•Primary health care centre			
•Trichiasis surgery facility [2]			
•Village pharmacy (drugstore)			
•Market			
•School			
•Other (specify) [3]			
•Notes and remarks:[3]			

1 Specify accessibility in terms of travel times to reach the nearest health facility.

2 If a mobile team visits the village from time to time to provide trichiasis surgery services, please make a note in the section "Notes and remarks".

3 Optional

Form 2

TRICHIASIS FORM

Village/community:
Population:

Entry number[1]	Patient's name[2]	Trichiasis[3]				Remarks
		Without CO4	Without CO4	Without CO4	Without CO4	
SUB-TOTAL		(A)	(B)	(C)		
TOTAL Trichiasis (a)+b)+(c)						
Village estimated prevalence (%)		(%)				

1. Insert a serial number.
2 Underline the name of persons who have agreed to be operated on.
3 Tick the box that applies.

Form 2 (Continued)

Notes and remarks:

4 CO (corneal opacity): easily visible corneal opacity over the pupil.
5 Recurrent case: person already operated on for trichiasis; however, at least one eyelash rubs on the eyeball.
6 Suspected case: person not examined by the team, but reported by family/neighbours to have eyelid/eyelash problem.

Form 3

ACTIVE TRACHOMA FORM

Village/community:

Name of head of household[1]	Child's name[2]	Active trachoma[3]		Unclean face[3]		Remarks
		TF	TI	YES	NO	

1 At least 20 households should be visited.
2 At least 50 children should be examined.
3 Tick the box that applies. Record the result of the worst eye.

Name of head of household[1]	Child's name[2]	Active trachoma[3]		Unclean face[3]		Remarks
		TF	TI	YES	NO	

Name of head of household[1]	Child's name[2]	Active trachoma[3]		Unclean face[3]		Remarks
		TF	TI	YES	NO	
TOTAL						
PERCENTAGE (%)						

Notes and remarks:

Form 4

ENVIRONMENTAL FACTORS FORM

Number[1]	Name of household head	Water source more than half an hour walk[2]		Presence of solid waste or animal pens[2]		Absence of functional latrine[2]	
		YES	NO	YES	NO	YES	NO

1 Give a unique identification number for each household visited.
2 Tick the box that applies.

Number[1]	Name of household head	Water source more than half an hour walk[2]		Presence of solid waste or animal pens[2]		Absence of functional latrine[2]	
		YES	NO	YES	NO	YES	NO
TOTAL							
PERCENTAGE OF «YES»							

Notes and remarks:

Form 5

RA SUMMARY SHEET

Village/community:

District: **Province:**

Assessment of trichiasis pattern	
NUMBER OF PERSONS EXAMINATED	.....
% OF TRICHIASIS	%
**Assessment of active trachoma pattern**	
NUMBER OF PERSONS EXAMINATED	.....
% OF CHILDREN WITH ACTIVE TRACHOMA	%
**Assessment of personal hygiene**	
NUMBER OF CHILDREN OBSERVED	.....
% OF DIRTY FACES	%
**Assessment of water availability**	
HOUSEHOLD MORE THAN HALF HOUR WALK FROM WATER SOURCE	%
**Assessment of proximity to garbage, human waster or animal pens**	
HOUSEHOLDS AT RISK	%
**Assessment of absence of latrine**	
HOUSEHOLDS WITHOUT LATRINE	%
